# Negative global phosphorus budgets challenge sustainable intensification of grasslands

**DOI:** 10.1038/ncomms10696

**Published:** 2016-02-16

**Authors:** S. Z. Sattari, A. F. Bouwman, R. Martinez Rodríguez, A. H. W. Beusen, M. K. van Ittersum

**Affiliations:** 1 Plant Production Systems Group, Wageningen University, PO Box 430, 6700 AK Wageningen, The Netherlands; 2 Department of Earth Sciences–Geochemistry, Faculty of Geosciences, Utrecht University, PO Box 80021, 3508 TA Utrecht, The Netherlands; 3 PBL - Netherlands Environmental Assessment Agency, PO Box 303, 3720 AH Bilthoven, The Netherlands; 4 Food and Agriculture Organization of the United Nations, Livestock Information, Sector Analysis and Policy branch (AGAL), Viale delle Terme di Caracalla, 00153 Rome, Italy

## Abstract

Grasslands provide grass and fodder to sustain the growing need for ruminant meat and milk. Soil nutrients in grasslands are removed through withdrawal in these livestock products and through animal manure that originates from grasslands and is spread in croplands. This leads to loss of soil fertility, because globally most grasslands receive no mineral fertilizer. Here we show that phosphorus (P) inputs (mineral and organic) in global grasslands will have to increase more than fourfold in 2050 relative to 2005 to achieve an anticipated 80% increase in grass production (for milk and meat), while maintaining the soil P status. Combined with requirements for cropland, we estimate that mineral P fertilizer use must double by 2050 to sustain future crop and grassland production. Our findings point to the need to better understand the role of grasslands and their soil P status and their importance for global food security.

## Abstract (web summary)

Grasslands lose soil fertility when manure from grazing livestock is spread on croplands. Here, Sattari *et al*. show that in order to achieve production increases that will meet global milk and meat demands for 2050, grassland phosphorus inputs must increase four-fold relative to inputs from 2005.

## Graphical abstract

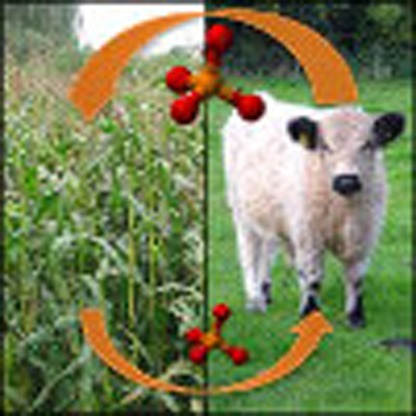

Global food demand will rise rapidly in the coming decades^1^,^2^,^3^. In particular, meat and milk consumption are projected to increase markedly due to rising incomes and decreasing prices^3^,^4^,^5^,^6^,^7^, especially in developing countries^8^. The global area of permanent grassland (3.3 billion hectares)^9^ covers 26% of the Earth's ice-free land, and provides an important contribution to global food security by supplying proteins and energy to ruminants. Between 1970 and 2005 the world's grasslands expanded by about 4%. Grassland degradation is likely to accelerate given the expected growth of livestock production^10^, overgrazing and consequent soil erosion, nutrient deficiency, weed encroachment and desertification.

Sustainably meeting global food demand is one of humanity's grand challenges^11^, and P is increasingly considered to be a new global sustainability issue because of its finite reserves^12^. Like nitrogen, P is a major limiting nutrient in agriculture^13^,^14^ and is removed from arable and grassland soils by crop and grass withdrawals and erosion. Soil P removed in the harvest needs to be replaced by weathering and inputs through organic (mostly manure) and mineral fertilizers to sustain crop and grass production^15^,^16^.

Most global studies on the agricultural P cycle focus on arable land^17^,^18^,^19^,^20^. However, we are not aware of any global study addressing the P cycle in grasslands and its connection with croplands. In this paper we assess grassland P budgets between 1970 and 2005, as well as P requirements to sustain projected future grass production. We consider soil P budgets to assess changes in soil fertility, and the budget for the grassland system to assess the P exchange between grasslands and croplands (Fig. 1). The classification of livestock production systems (intensive and pastoral) and grassland systems is shown in Supplementary Fig. 1.

This study focuses on P, but it is evident that nutrient management will have to consider all nutrient requirements in grazing systems, including nitrogen, potassium and many other (micro-) nutrients.

Our general conceptual framework for P budgeting in grasslands focuses on grazing systems including the key P inflows and outflows of four compartments within the grassland system boundary: grassland-based livestock population, grassland-based livestock manure, soil (including weathering) and grass (Fig. 1). Our calculations include all countries of the world. We distinguished two categories of grasslands, that is, those occurring in mixed (and landless) livestock production systems (denoted as intensive systems) and those in pastoral systems. The difference between intensive and pastoral systems is in the P exchange between grassland and croplands through feed and manure. Most pastoral systems are in developing countries, and typically there is little exchange of nutrients between these pastoral systems and croplands (little feed import or manure export). However, there is P withdrawal in the various products (milk, meat, wool, skin and so on). The intensive ruminant systems are mixed, that is, they rely on grass, often supplemented with feed produced in croplands, and part of the manure produced in these systems is collected (in ‘kraal' or animal houses) and spread in the backyard farms or croplands. Where P in manure exported from grassland thus exceeds the P in feed, there is net P transfer from grassland to cropland. The pastoral systems are extensive everywhere and generally located in dry or cold climates in remote regions away from human populations and croplands. In contrast, the intensive (mixed) systems generally feature in more favourable climates, but their productivity varies from country to country; in industrialized countries they are generally intensive with high productivity of grasslands and animals and large feed requirements, and they are less intensive in developing countries (with limited feed use, often low-quality feed such as crop residues, and relatively low grass and animal productivity).

Using the Dynamic Phosphorus Pool Simulator (DPPS)^15^ model we simulated historical P uptake in grasslands and then calculated the amount of P needed for meeting the global grass requirement in intensive and pastoral systems until 2050 according to the Rio+20 scenario, avoiding soil P deficits and surpluses^21^. This research is the first study that estimates soil P budgets in grasslands at the global scale and hence allows the assessment of total future P requirements of agriculture. The P budget of the grassland system was found to be negative between 1970 and 2005 in most of the world regions. Thus, to meet anticipated growth of ruminant production and associated grass requirement, the nutrient status of grassland soils needs to be improved to increase productivity of existing grasslands and to avoid expansion of grassland areas. We estimate that P inputs (mineral and organic) will have to increase more than fourfold in 2050 (relative to 2005) to meet the projected demand for ruminant products as targeted in the Rio+20 (ref. 21) scenario for 2050.

## Results

### Agronomic soil P budget

Historical data show that soil P uptake by grass in intensive and pastoral grazing systems exceeded P application (input) through fertilizer and manure in most parts of the world (Fig. 2). Both uptake and applications were relatively low during the period 1970–2005 in most regions of the world (≈5 kg ha^−1^ per year or less) in contrast to the more intensive grazing systems of Western and Eastern Europe (Fig. 2). Specifics for the intensive and pastoral systems are shown in Supplementary Figs 2 and 3, respectively.

Between 1970 and 2005 cumulative inputs of P in Eastern Europe (38 Tg P; Tg=teragram=10^12^ g) and Western Europe (56 Tg P) were slightly larger than the cumulative grass uptake of 34 and 49 Tg P, respectively; North America and Oceania had a balanced P budget. By contrast, cumulative soil P uptake by grass in Africa (76 Tg P), Asia (191 Tg P) and Latin America (96 Tg P) exceeded P inputs, particularly in Asia (65, 72 and 82 Tg P, respectively) over the same period (calculations are based on Fig. 2 and grassland area shown in Table 1).

Manure was the most important input of P for grassland soils worldwide and mineral fertilizer P application was systematically much smaller than P in manure in all world regions (Table 1). During the 1970–2005 period only 23 Tg of mineral fertilizer P was applied to global grassland soils, which is similar to the global use of mineral P fertilizers in croplands of 18.5 Tg P in just the single year of 2010 (ref. 22). Europe alone accounted for almost 80% of the global cumulative mineral P fertilizer use in grassland.

### Grassland system P budget

**Figure 1:**
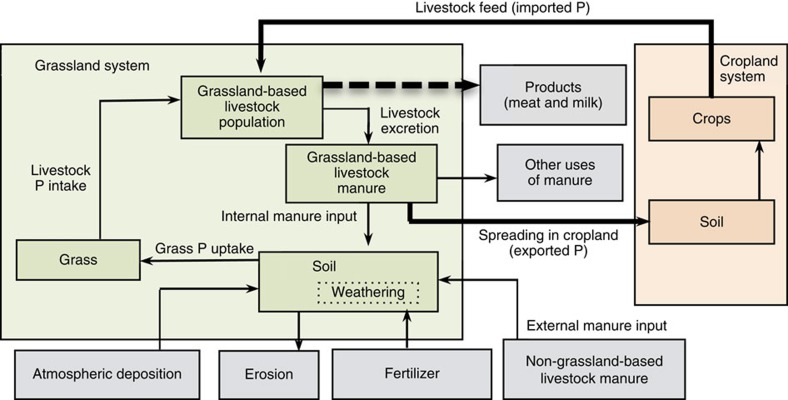
Phosphorus budget model for the grassland system. Phosphorus flows within grassland and between the grassland and cropland systems. The grassland system comprises four compartments (grassland-based livestock population, grassland-based livestock manure, soil including weathering supplying P from soil minerals and grass) and six compartments outside the grassland boundaries (products, other uses of manure, erosion and atmospheric deposition, fertilizer (only in the intensive system) and non-grassland-based livestock manure). Next to the grassland system budget, we also consider the agronomic soil P budget, with the soil surface as boundary; grass P uptake (that is, the P withdrawal by grass harvest or grazing) is considered as output, and mineral fertilizer and manure (internal and external manure inputs) as inputs. We assume that grass P uptake equals livestock P intake. Thus, this approach ignores any P losses during mowing, transporting or stall-feeding of grass. P transfers between grasslands and croplands are represented by the flows (thick arrows) ‘Livestock feed' (imported P from cropland to grassland as livestock feed) and ‘Spreading in cropland' (exported P, the manure P that is deposited in grasslands, but is transferred to croplands for spreading). The bold, dashed arrow represents the P flow leaving the grassland systems via animal products, mainly meat and milk.

**Figure 2:**
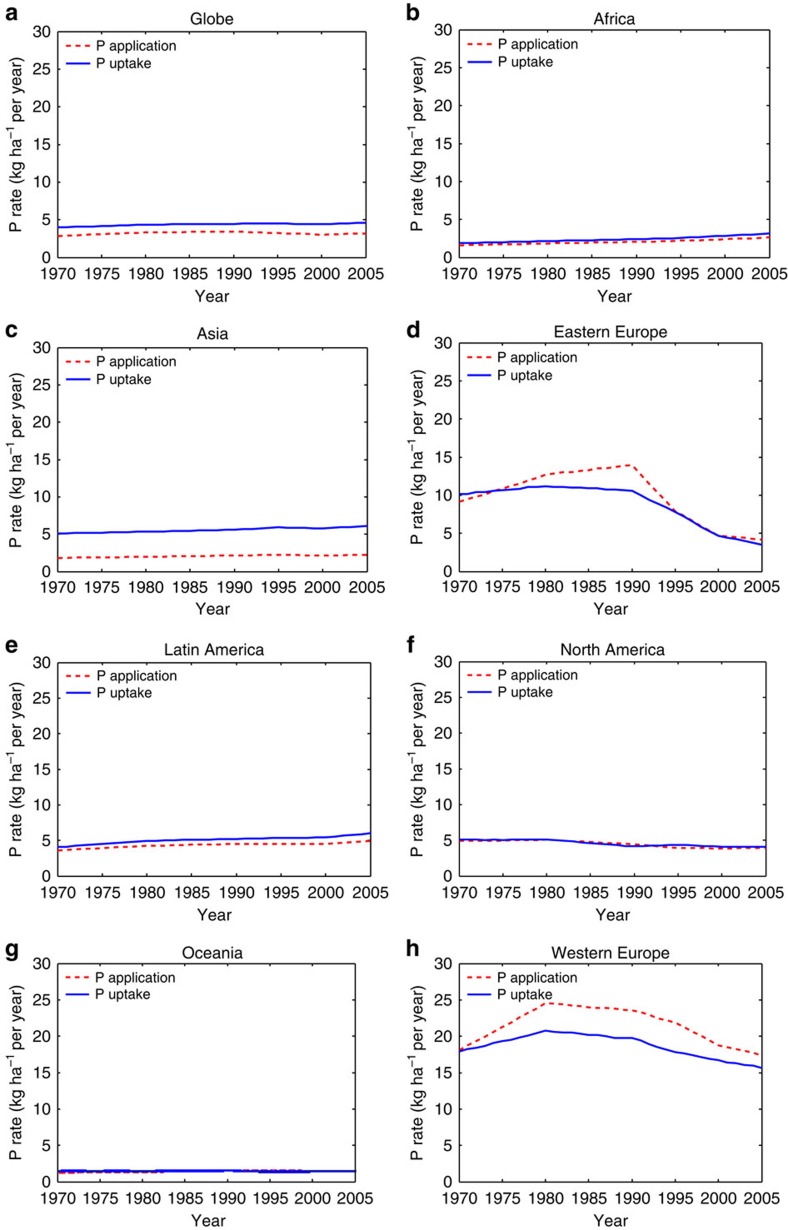
Agronomic soil P budget. Historical trends of annual P application and P uptake in grassland systems (intensive and pastoral) for the period 1970–2005 in (**a**) Globe, (**b**) Africa, (**c**) Asia, (**d**) Eastern Europe, (**e**) Latin America, (**f**) North America, (**g**) Oceania and (**h**) Western Europe. These regions were also used in a previous study on residual P in cropland^15^. Dashed and solid lines represent P application and P uptake, respectively. P application represents the P inputs from manure plus mineral fertilizer to the grassland soils and P uptake refers to grass P uptake.

Manure P excretion is an input in the soil P budget (Fig. 1), but in the grassland system it is in fact partly an internal cycling of P, as it partly originates from grass P uptake from the soil. Manure P application from non-ruminants (external manure), fertilizer P, atmospheric P deposition and P in imported feed are considered grassland system P inputs and manure P exported from grassland to cropland, P that leaves grasslands via livestock products, P in the other use of manure (for non-agricultural purposes) and erosion are included as grassland system P outputs.

The total P budget of the grassland system was negative in 1970 and 2005 in most of the regions; only Eastern Europe and Oceania had (small) P surpluses (Table 1). Globally, grassland systems received an estimated 125 Tg of P (from external manure, mineral P, atmospheric deposition and feed) between 1970 and 2005. Over the same period, estimated global cumulative P outputs (P in manure exported to cropland, livestock products, the other use of manure and erosion) were 264 Tg P, which caused a negative P budget of −139 Tg for grassland systems between 1970 and 2005.

### Phosphorus transfers between grasslands and croplands

**Figure 3:**
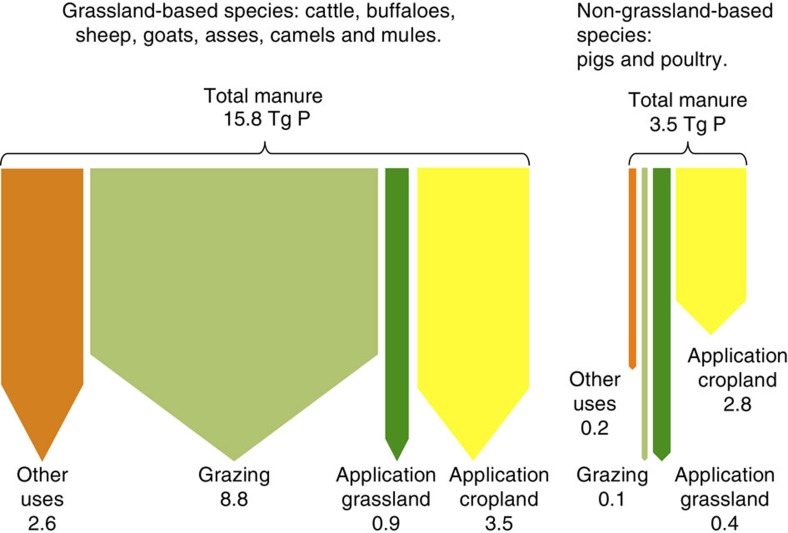
Manure allocation framework. The figure illustrates the manure allocation framework and the global data for 2005. All flows are shown in Tg of P per year. ‘Other uses' represents the use of manure for non-agricultural purposes such as fuel. ‘Grazing' and ‘Application grassland' represent the amount of manure deposited as animals graze and the amount that is spread as organic fertilizer in grasslands, respectively. Both are accounted as grassland soil P inputs. ‘Application cropland' is the amount of manure used as organic fertilizer in croplands.

Two flows are involved in the P transfers between grassland and cropland systems, that is, ‘Livestock feed (from croplands)' and ‘Spreading (of manure) in croplands' (Figs 1 and 3). On the one hand, grassland-based ruminants consume feed produced in croplands, which effectively imports P into the grassland systems. On the other hand, P is effectively transferred from grasslands to croplands when manure from ruminants is used as organic fertilizer in the latter systems.

**Figure 4:**
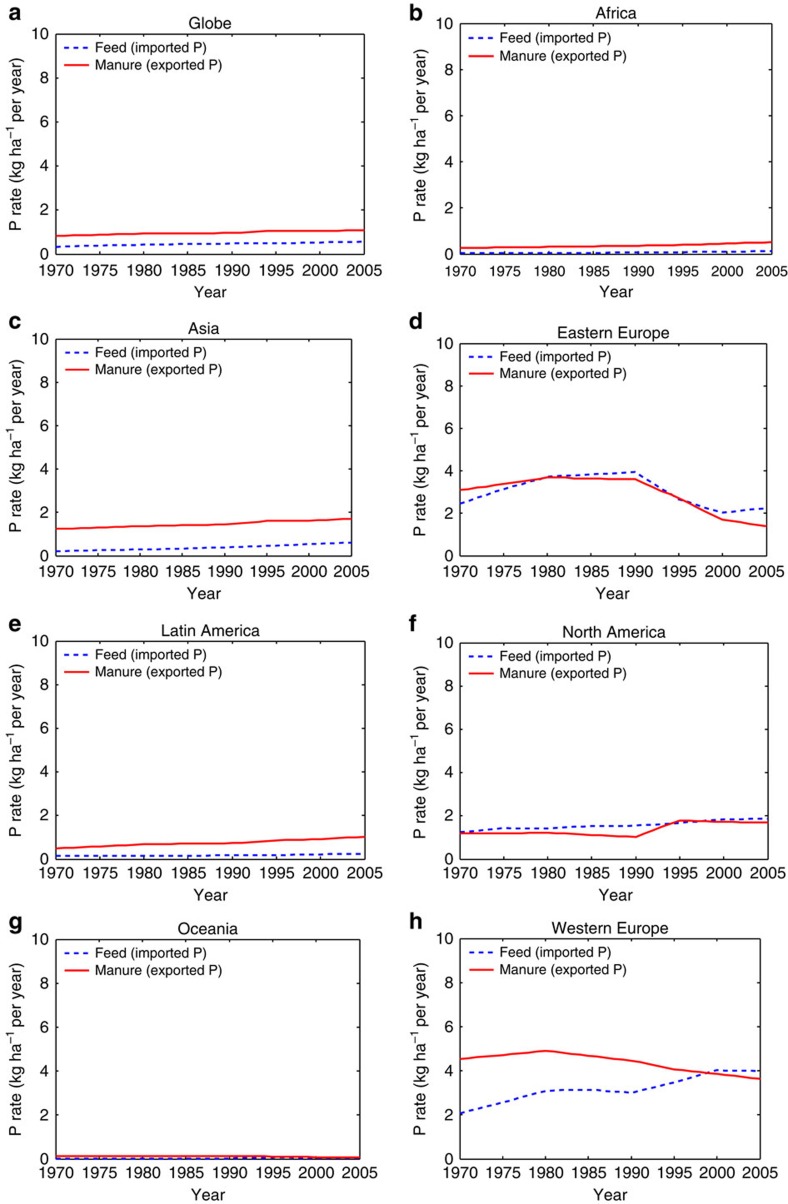
Phosphorus transfers between grasslands and croplands. Historical trends of annual P import to and export from grasslands (intensive and pastoral) as livestock feed and manure spread in croplands, respectively, for the period 1970-2005 in (**a**) Globe, (**b**) Africa, (**c**) Asia, (**d**) Eastern Europe, (**e**) Latin America, (**f**) North America, (**g**) Oceania and (**h**) Western Europe. Dashed and solid lines represent imported P (feed) and exported P (manure), respectively. Imported P stands for the feed produced in croplands that is consumed by livestock on grasslands, and exported P stands for the manure P that originates from grasslands (intensive and pastoral), but is transferred to croplands.

Feed use varied between different world regions (Fig. 4). Africa and Oceania had a maximum rate of feed use of only 0.1 kg P per ha per year in 2005 (Table 1), while Western Europe's feed use (4 kg P per ha per year) was around seven times the global rate (0.6 kg P per ha per year) in 2005 (Table 1 and Fig. 4a). The feed P import to the grassland systems in Asia and Latin America remained minimal at 0.7 and 0.2 kg P per ha per year, respectively, in 2005. North America's feed input rate was 1.9 kg P per ha per year in 2005 (Table 1 and Fig. 4).

North America (with 14.5 Tg), Asia (13.3 Tg), Eastern Europe (11.7 Tg) and Western Europe (8.2 Tg) were responsible for about 90% of cumulative global feed use (53.4 Tg) in 1970–2005. In contrast, the imported P from croplands to grassland (as livestock feed) in Latin America (3.4 Tg), Africa (2.1 Tg) and Oceania (0.4 Tg) accounted for only 10% of the global total (1970–2005).

In 1970, the application of manure to cropland soils varied from 0.1 kg P per ha per year in Oceania to 4.5 kg P per ha per year in Western Europe (Table 1 and Fig. 4g,h). The maximum manure P export to croplands occurred in 1980 in Western Europe (4.9 kg P per ha per year) followed by Eastern Europe (3.7 kg P per ha per year). The other world regions reached rates of only 1.8 kg P per ha per year (Asia) and 1.7 kg P per ha per year (North America) or less in 2005. Cumulative global use of manure as fertilizer in croplands for the 1970–2005 period was 113 Tg of P. Asia alone was responsible for 44% of the global number, with a total of 50 Tg P. Africa (11.7 Tg P), Eastern (11.3) and Western Europe (11.5) had an almost equal share in the global P export from grassland to cropland (≈10% each). Cumulative transfer of P to cropland (1970–2005) was only 1.7 Tg in Oceania, 14.3 Tg in Latin America and 12.6 Tg in North America.

Historical trends of annual P feed imports to grasslands and manure exports from grasslands to croplands for the intensive grassland system for the period 1970–2005 are shown in Supplementary Fig. 4.

### Future demand of P in grassland

**Figure 5:**
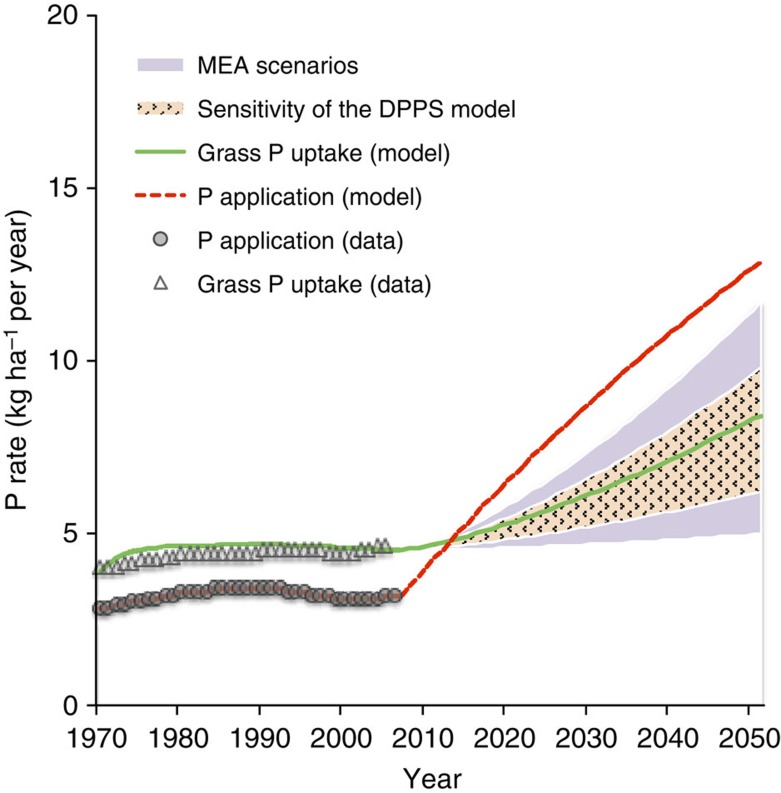
Trends of global annual P application and grass P uptake in grassland for the period 1970–2050; the 2050 target uptake was derived from the Rio+20 scenarios. The uncertainty in the target 2050 uptake of 8.3 kg P per ha may amount to ±40% (based on the difference between the four Millennium Ecosystem Assessment (MEA) scenarios^28^—shading). According to the model sensitivities, the variation in the simulated uptake as a result of variation of all the parameters for 2050 results in −26 to +17% (2.5 and 97.5 percentiles) around the median of 8.3 kg ha^−1^ (dot shading). Markers and lines illustrate long-term historical data and simulation results, respectively. Circles and triangles refer to P application and P uptake rates, respectively. Dashed red and solid green lines refer to P application and P uptake rates, respectively. P application stands for P inputs into the soil including internal manure, external manure and fertilizer. P uptake stands for the grass P uptake. The *R*^2^ value for calculated (model) versus observed (data) P uptake (1970–2005) is 0.65.

We used the DPPS model^15^ to estimate P application rates (mineral fertilizer and manure) needed to achieve projected future grass production under the Rio+20 scenario^21^. Rio+20 projects a substantial increase in required global grass production and P uptake between 2005 and 2050 from 4.6 to 8.3 kg per P per ha due to the rapid increase in global ruminant meat and milk consumption and production (Fig. 5). This increase in grass production can come from either expansion of grasslands or enhanced productivity. Here we assume no expansion of the grassland area, so raising grass production will only be possible if the P status of soils under grassland is improved. With this assumption, the DPPS model estimates that to achieve the 2050 target grass production, 375 kg P per ha is required between 2006 and 2050, resulting in a cumulative global P requirement of 1,220 Tg (Fig. 5). According to the Rio+20 scenario, 650 Tg of this cumulative P input can be supplied through manure. To achieve the target grass production and P uptake until 2050 and assuming no expansion of grasslands, the rest of the required cumulative P input (570 Tg) needs to come from mineral P fertilizer. The 2.5–97.5 percentiles of the required P uptake in global grasslands under the Rio+20 scenario are, respectively, 810 and 1,500 Tg, for the period 2006–2050.

## Discussion

This paper presents the first estimates of global and regional soil P budgets in grasslands. In terms of production, mixed and landless systems are dominant for all ruminants (73% of beef, 92% of milk, 65% of mutton and goat meat and nearly all monogastrics production); however, in terms of area usage, the pastoral systems are covering more than 80% of the global grassland area^23^. Although the contribution of grass to total dry matter intake by all animals is projected to decrease from 50 to 45% between 2005 and 2050 (Rio+20 data), the total amount of grass needed for the increased livestock production will increase substantially. Assuming no area expansion, this means that grasslands will need improved nutrient management, with inputs of P in balance with N and other nutrients.

The share and absolute amounts of P in manure transferred from grassland to croplands are much larger for intensive than for pastoral grassland systems (Table 2). The estimated cumulative global use of manure originating from grassland to fertilize croplands during the 1970–2005 period was 113 Tg of P, Asia alone being responsible for 44%. For instance in China, the grazing system was confronted with a severe P deficit problem due to the massive transfers of P in the form of manure, while grasslands were hardly fertilized^24^,^25^. North America and Eastern Europe were the only regions that showed a cumulative net P transfer from cropland to grasslands, that is, 1.9 and 0.4 Tg P, respectively (calculated from Fig. 4). Our estimations for the different P flows generally show a fair agreement with values reported in the literature.

P in ruminant meat and milk represents a small amount compared with P in the manure, reflecting the low P conversion efficiency in ruminant systems. Although soil stocks of P in grasslands are substantial^26^, there is a systematic loss of soil fertility through export in manure to cropland that may aggravate current rates of soil degradation across most of the global grasslands. More grass production can be achieved, while avoiding grassland expansion and deforestation, through mineral P fertilization (and maintaining a balance between nitrogen, P and potassium^13^,^27^), adequate management of livestock and manure, and soil conservation to control P loss through erosion. More ruminant production in intensive systems based on crop feed concentrates, or less manure transfer from grasslands to croplands, will both require more P use in the world's croplands.

Our estimate of the global P input required in grasslands of 1,220 Tg P for the 2005–2050 period includes P from both mineral fertilizer and animal manure. In 2050, globally 24 Tg per year of mineral P fertilizer will be needed to avoid loss of soil fertility and declining grassland productivity. Sattari *et al*.^15^ estimated that to increase crop production by 55% (this is the estimation based on the Global Orchestration scenario of the Millennium Ecosystem Assessment (MEA)^28^) in 2050 relative to 2005, P from mineral and organic fertilizer has to increase by only 20% due to the so-called residual soil P effect. Thus, excluding the share of manure P (32%) from the total required P, the estimated global mineral fertilizer P requirement in croplands was 20.8 Tg per year in 2050 (ref. 15). This indicates significant opportunities to increase crop production, with less than proportional increases of P use and associated environmental impacts. By contrast, we estimate here that to realize an anticipated 80% increase in grassland production (for milk and meat), the P input from fertilizer (mineral and organic) will have to increase fourfold. Thus, the amount of mineral fertilizer P needed in cropland and grassland systems in total is estimated to be 45 Tg per year in 2050, corresponding to 350 Tg phosphate rock. For the entire agricultural system (crop and grass production), we estimate that relative to 2005 mineral P fertilization needs to more than double by 2050. In the next four decades, a global cumulative amount of mineral fertilizer P of 1,390 Tg is required, accounting for 820 Tg P for cropland soils^15^ (2008–2050) and 570 Tg P for grassland soils (2005–2050), to achieve the projected global food production in 2050. This implies that up to 2050, 10,700 Tg of phosphate rock will need to be mined across the globe to produce the P fertilizer for the agricultural and food production sectors.

Livestock grazing systems are far more complex than crop production systems, and the available data on grass production are scarce (see Methods section). For example, Food and Agricultural Organization provides livestock production, animal stocks, numbers of slaughtered animals and milking cows, carcass weights and milk yield per animal^22^, but lacks data on the age distributions of animals and more importantly, data on grass production and management and grass withdrawal by grazing and mowing. Similarly, most national statistics lack such data^29^,^30^. Our sensitivity analysis revealed that the global model results for the total grazing system are strongly sensitive to animal stocks, excretion rates and the initial labile and stable soil P pools, which are known to be uncertain^31^ (Supplementary Table 8). Yet, our modelling results and data analysis suggest that negative P budgets in the world's grassland soils can be an obstacle when intensifying grassland production. This comprehensive analysis underlines the necessity to adopt nutrient management strategies, balancing P, nitrogen and other nutrients for croplands and grasslands. It also shows the urgent need for empirical data on grass production, fertilization and nutrient withdrawal.

## Methods

### Overview

We coupled two models in our study, that is, the soil P budget model (Fig. 1) and the DPPS model, to reproduce the soil P budget for the 1970–2005 period and to estimate future P requirements in grasslands. The soil P budget model considers two system boundaries: the grassland system boundary and the grassland soil boundary. Phosphorus is leaving the grassland systems' boundary via animal products (mainly meat and milk), livestock manure and runoff or erosion. On the other hand there is P import from cropland into grassland through animal feed.

Within the grassland soil boundary, we distinguish different sources of soil P inputs, including manure generated inside the grasslands and spread in the grasslands (grassland-based, internal, livestock manure; Fig. 1), manure produced by pigs and poultry outside the grassland boundaries (non-grassland-based, external, livestock manure; Fig. 1), mineral P fertilizer (only in intensive livestock production systems, see section below) and P from weathering and atmospheric deposition. Grass P uptake, runoff and erosion are regarded as P outflows from the grassland soil (Fig. 1). Manure generated within the grassland boundaries is assigned to three uses: spreading in grassland soil, spreading in croplands and other uses of manure. The net transfer of P between croplands and grasslands over the period 1970–2005 is the difference between P imported to grassland from cropland through livestock feed, and P export from grassland to cropland in the form of manure (Fig. 1 and Supplementary Fig. 1). The DPPS model is presented in detail below.

All abbreviations used in the soil P budget model are explained in Table 3, and Table 4 describes the P flows, the animal categories and the data sources. The data used in this paper as well as the DPPS model are available on: http://models.pps.wur.nl/content/DPPS-Grassland.

### Livestock production systems and animal groups

In their global classification, Seré and Steinfeld^32^ distinguished livestock production systems (LPSs) and mixed farming production systems (MPS). LPS includes systems in which more than 90% of dry matter fed to animals comes from rangelands, pastures, annual forages and purchased feed. MPS are systems in which more than 10% of the dry matter fed to animals comes from crop by-products or stubble, or more than 10% of the total value of production comes from non-livestock farming activities^32^. They further distinguished four subgroups: landless LPS; grassland LPS; rainfed MPS and irrigated MPS (Supplementary Fig. 1a). They collected data on buffaloes, cattle, goats, pigs, poultry and sheep population numbers and meat and milk production in each system. Later, Bouwman *et al*.^23^ modified the classification from Seré and Steinfeld^32^ for their global analysis of ruminant production systems. Landless LPS and MPS were merged into mixed-landless systems (or intensive systems) while grassland LPS was renamed into pastoral systems (Supplementary Fig. 1b,c).

Following Bouwman *et al*.^23^, in the present study also two production systems were distinguished, that is, grasslands in mixed and landless (referred to as intensive hereafter) and extensive pastoral LPSs. Within each system, two groups of animals were considered: ‘grassland-based livestock' including asses, buffaloes, camels, dairy cattle, horses, mules, non-dairy cattle, sheep and goats, and ‘non-grassland-based livestock' including pigs and poultry (Fig. 3). Owing to lack of data, it was not possible to include all animal categories for all the calculations (Table 4). Furthermore, non-grassland-based livestock categories were not included in products' P flow calculations since in our definition they are not located within the grassland system boundaries (Fig. 1).

### Data used

The P flows considered for different animal categories and their data sources are listed in Table 4. Phosphorus inflows to the grasslands comprise feed, P fertilizer, external manure P and atmospheric deposition; P outflows from grasslands boundaries include P in livestock products, exported manure to croplands, other use of manure P and P in runoff or erosion.

Long-term livestock production data (meat and milk) were obtained from the Integrated Model to Assess the Global Environment (IMAGE)^33^, which is based on FAOSTAT^22^ data; the average P content of meat and milk and various animal parts was obtained from the literature^23^.

The animal stocks within pastoral and intensive livestock production systems and their P excretion rates were used to calculate the total P in manure within the two systems (see section on livestock manure P).

The amount of feed for each country, year and animal category was estimated based on IMAGE^33^ output data and on the use of grass and feed crops and energy requirements of the different animal categories).

The total mineral P applications to grasslands in mixed and landless livestock production systems for 1970–2005 were from IMAGE at the country scale, based on FAOSTAT and the International Fertilizer Association^34^,^35^).

Weathering and atmospheric deposition as P inflows to grasslands and soil P erosion and runoff as P outflows were estimated from literature review and IMAGE output data. The sections below provide more details on the methods and data used for the various model components.

### Livestock products

Livestock production data include milk, meat and livestock by-products. Livestock by-products include adipose tissue, skeleton, viscera, blood, skin, hair and digestive content. Country-scale meat production data for non-dairy cattle and sheep and goats, as well as milk production were obtained from FAOSTAT^22^. Meat and milk production for buffaloes, horses, asses, mules and camels was not included due to lack of data. The total amount of P in livestock products is the sum over P in milk, meat and livestock by-products.

The carcass weight is the most common way of expressing livestock meat production. Carcass weight is defined as the weight left after slaughter and removal of head, skin, genitourinary organs and offals. The ratio between the carcass weight and the live weight is called the dressing percentage (DP). Live weight consists of the following fractions: muscles; adipose tissue; skeleton; viscera; blood; skin; hair; and digestive content. Live weight partitioning allows for a more accurate P accounting due to large differences in P concentration in different fractions such as bones and blood.

To allocate the total national production to mixed-landless and pastoral systems, national, annual values of the fraction allocated to mixed-landless systems (Fraction intensive) were obtained from IMAGE^33^. A list of countries and regions as used in the present study based on IMAGE has been shown in Supplementary Table 1.

In addition, data on DPs were obtained from Kempster *et al*.^36^, who reported 53 and 50% DP for, respectively, non-dairy cattle and for sheep and goats. Live weight partitioning fraction (LWF) data and P content of those fractions were obtained from different sources (Supplementary Table 2).

Equation (1) was used to calculate P in meat and livestock by-products:


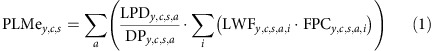


where *y* denotes year, *c* country, *s* production system, *a* animal category and *i*, fraction of meat and by-products.

Phosphorus in meat and livestock by-products is denoted by PLMe (kg P). LPD and DP refer to the livestock production data (kg carcass weight) and DP (kg carcass per kg live weight), respectively. LWF (kg meat or livestock by-products per kg live weight) represents live weight partitioning and FPC refers to P content (kg P per kg meat or livestock by-products).

The total amount of P in milk was calculated by multiplying the total amount of milk production with the P content of milk, as shown in equation (2). The P content was considered to be 8.4·10^−4^ kg P per kg milk^30^.


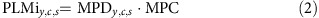


where *y* denotes year, *c* country and *s* production systems.

Phosphorus in milk is expressed by PLMi (kg P). MPD and MPC refer to the milk production data (kg milk) and milk phosphorus content (kg P per kg milk), respectively.

### Livestock manure phosphorus

Total manure production within pastoral and intensive systems was computed from animal stocks and P excretion rates. We used P excretion rates per head for dairy and non-dairy cattle, buffaloes, sheep and goats, pigs, poultry, horses, asses, mules and camels based on various sources^37^,^38^,^39^,^40^,^41^ (Supplementary Table 3). We used constant excretion rates per head; in case of increasing production per head, fewer animals are needed to produce the same amount of meat or milk; total P excretion will thus decrease, and the excretion per unit of product decreases reflecting increasing conversion efficiency due to improved feeding and management. For each country, animal stocks and P in the manure for each animal category were spatially allocated across intensive and pastoral systems. For the time period 2005–2050, the distribution over these systems is provided by the Rio+20 study^21^.

The methodology and equations described in this section were applied to all animal categories.

The manure allocation comprises five steps: a first calculation of total excreted manure and its P content and a subsequent fractioning into four different flows (Fig. 3).

The total amount of manure P was calculated according to equation (3).


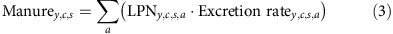


where *y* denotes year, *c* country, *s* production systems and *a* animal category).

‘Manure' refers to the total P in manure excreted by livestock (kg P); LPN is livestock population (number of heads); ‘Excretion rate' is the annual amount of P excreted for each animal category (kg P per head per year). The sum of manure for grassland-based animal categories (cattle, buffaloes, sheep, goats, camels, horses and asses) corresponds to the ‘Livestock excretion flow' depicted in Fig. 1.

The amount of P (kg) in manure used for non-agricultural purposes (other uses) was calculated according to equation (4).


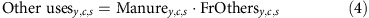


‘FrOthers' expresses the fraction of manure used in any way so that it is effectively removed from agricultural systems, as it could be used as fuel or building material, or digested for generating energy.

From the remaining manure, the amount of P (kg) in the manure allocated to grazing was estimated by equation (5).


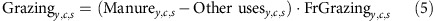


‘FrGrazing' refers to the fraction of manure that is deposited in grasslands by grassland-based grazing animals. In the case of non-grassland-based species, it represents their deposition as they scavenge in grasslands.

The rest of the manure P was considered to be excreted in animal houses and stored, and available for use as organic fertilizer either in grasslands or croplands. Two fractions were calculated, ‘Grasslands' and ‘Croplands' as shown in equations (6) and (7), respectively.


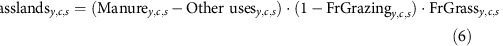


‘FrGrass' refers to the fraction of stored manure that is applied in grasslands as organic fertilizer.

The amount of P (kg) in the manure that is transferred from animal houses or stored manure to cropland as fertilizer was estimated by equation (7).


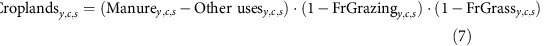


The sum of ‘Grazing' and ‘Grasslands' for grassland-based and non-grassland-based species corresponds to the internal and external manure input flows, respectively, as depicted in Fig. 1.

The values for the manure allocation fractions (other uses, grazing and application to grasslands) are listed in Supplementary Table 4.

### Livestock feed

The IMAGE model^33^ used FAOSTAT data on crops for feed and energy requirements of animals to estimate the amount of feed for each country, year and animal category. Only the animal categories of non-dairy cattle, dairy cattle and sheep and goats were considered for estimating feed crop use. Thus, a 100% grass ration was assumed for the rest of grassland-based animal categories (buffaloes, horses, asses, mules and camels).

IMAGE provides feed data for 11 feed items: oil crops; maize; pulses; rice; root and tuber crops; temperate cereals; tropical cereals; crop residues; and other by-products such as residues from breweries, and grass and scavenging (such as road-side grazing, food waste and so on). FAOSTAT provides data on the amount of feed crop use per country, but does not specify feed use by animal category. In IMAGE the specification of feed crop use for individual animal categories is based on feed rations that are calibrated to match FAOSTAT data on total feed use at the scale of world regions. Therefore, disaggregation of world-region feed crop use by animal category to the country scale was needed. To do so, it was assumed for each animal category that the fraction of feed crops in the ration is proportional to livestock productivity. The total amount of feed crops available for a specific animal category within a world region was thus allocated on the basis of the productivity of the animal category considered in a specific country relative to the regional productivity. Country and regional productivity were calculated for each animal category as the country and regional amount of product divided by the country and regional animal numbers, respectively. Since this calculation was done for each animal category, productivity numbers were expressed in terms of kg of carcass weight per head (for non-dairy and sheep and goats) or kg milk per head (for dairy cattle). Then, for each country the ratio between its productivity over the regional productivity was computed, which yielded a weighing factor (first term of equation (8)). With the information on the regional feed amount and the animal numbers, the regional average feed intake was calculated for each feed item and animal category. The total amount of P for each feed item for a specific animal category within a certain country was thus the product of the weighting factor times the country's animal numbers times the regional average feed intake times the P content of the feed item. By adding up all the feed items and animal categories, the country total P amount in livestock feed crops was calculated.


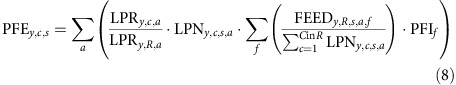


where *y* denotes year, *c* country, *s* production systems, *a* animal category, *f* feed item and *R* regional data).

PFE (kg P) is total amount of P in livestock feed. LPR refers to the livestock productivity (kg per head). It is calculated as the total amount of products associated with the animal category (carcass weight or milk) over the total number of animals. LPN refers to the animal numbers and the total amount of feed item used in a certain region as presented by FEED (kg). PFI (kg P per kg feed item) is the P content for a given feed item.

It is clear that part of the P in feed used in grassland-based systems is imported from other countries. Owing to lack of data we ignore feed trade, but in regions such as Western Europe, P in feed produced in croplands in other world regions may be a significant part of total feed use. However, this does not affect the global budget of the grassland-based systems, but may lead to overestimation of the net transfer from cropland to grassland within feed importing countries.

### Livestock grass intake and grass P uptake

The estimation of grass uptake (and grass intake by animals) is based on a mass balance approach. At the animal level, P inputs (feed plus grass intake) equal the P outputs (manure plus products). Phosphorus in the livestock grass intake was assumed to be equal to grass P uptake from the soil. Thus, this approach ignores any P losses during mowing, transporting or stall-feeding of grass. The grass P uptake was calculated according to equation (9). The amount of feed (PFE) and products (PLMe and PLMi) were assumed to be zero for buffaloes, horses, asses, mules and camels. For these animal categories the amount of P in grass intake equals the excretion of P.


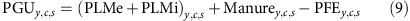


where *y* denotes year, *c* country and *s* production systems.

PGU (kg P) is the total grass phosphorus uptake and PFE (kg P) refers to the total amount of phosphorus (kg) in livestock feed.

### Mineral phosphorus fertilization

The IMAGE data include the total use of mineral phosphorus fertilizers in grasslands for 1950, 1970, 1980, 1990, 1995, 2000 and 2005 at country level. The values are based on FAOSTAT^22^ and the International Fertilizer Industry Association^34^,^35^ for the period 1970–2000, and extrapolations for earlier and later years^4^,^21^. Those numbers were exclusively allocated to the mixed and landless production systems, as we assumed no use of mineral fertilizer in pastoral systems.

### Soil phosphorus erosion

Soil P loss estimates were based on a recent modelling approach, which distinguishes two nutrient loss pathways to estimate P runoff and erosion^42^, that is, losses from recent nutrient applications (*P*_rec_) in the form of fertilizer, manure or organic matter^43^, and a residual or ‘memory' (*P*_mem_) effect related to long-term historical changes in soil nutrient stocks for the top 30 cm (refs 44, 45). The approach presented by Cerdan *et al*.^46^ based on a large database of measurements was used as a basis for calculating P_mem_ based on slope, soil texture and land cover type. This approach yields an average soil loss of 40 tonnes of soil per km^2^ year for Europe^46^.

*P*_rec_ was calculated from P inputs and depends on slope (using the approach of Bogena *et al*.^47^) and was further modified by land use and soil texture, that is, those factors that reduce surface runoff according to Velthof *et al*.^48^,^49^

The initial P stock in the top 30 cm was taken from Yang *et al*.^50^ for the year 1900, and residual soil P was calculated from the soil P budget up till the year 2005 (ref. 42). All inputs and outputs of the soil balance were assumed to occur in the top 30 cm; the soil budget model replaces P enriched or depleted soil material lost at the surface by erosion with fresh soil material (with the initial soil P content) at the bottom.

Simulated cumulative P loss amounted to 33 Tg P over the 1970–2005 period, which is about 1 Tg P per year or on average 30 kg P per km^2^ per year. For a mean P content of soils of 0.5 kg per tonne of soil^51^,^52^ this means a soil loss of 60 ton km^−2^, which exceeds the European estimates by 50% due to larger erosivity of grasslands in especially tropical and (semi-)arid climates.

Estimates of erosion rates are uncertain. Erosion rates in overgrazed grasslands have been estimated to amount to 1,500 t of soil per km^2^ per year^52^,^53^. Such rates may locally be possible, but as a global average they seem unrealistic as they exceed the European average erosion rates of 40 t of soil per km^2^ per year^46^ by a factor of 40.

### Weathering and atmospheric deposition

On the basis of the ratio of cropland area to world total land areas, Liu *et al*.^52^ calculated the amount of weathering and atmospheric deposition on croplands. Employing the same approach, we have estimated about 4.0 Tg P per year as weathering and atmospheric deposition in grasslands.

### Scenario for the period 2006–2050

There are different global scenarios, such as from the Organization for Economic Cooperation and Development^54^, MEA^28^, IPCC-SRES^55^ and Rio+20 (ref. 21). For this study, we needed scenarios that focus on nutrients and included future food demand, production and the nutrient uptake. Particularly future production and consequently P uptake was needed as a target for selecting the appropriate scenarios. A few recent examples of scenarios that included projections of nutrient uptake are the MEA^28^ and more recently the Rio+20 (ref. 21) scenarios.

Future demand of P fertilizer in global croplands was calculated based on the target crop yields, given by four different MEA scenarios for 2050 (ref. 15).

Recently, van Vuuren *et al*.^21^ developed new pathways (Rio+20) to achieve global sustainability goals for food, land and biodiversity, as well as for energy and climate by 2050. The Rio+20 study describes four scenarios, that is, the Trend scenario and three challenge pathways. The Trend scenario describes possible trends in the absence of climate and sustainability policies. The three challenge pathways were designed to assess the potential to achieve sustainability goals.

We used the Rio+20 (ref. 21) Trend scenario for simulating future P requirement in grasslands. Baseline scenarios represent a continuation of current trends, with no marked changes or shifts in production and management systems, attitude towards environmental problems and so on. The Rio+20 Trend scenario is a baseline or business-as-usual scenario, and comparable with the baseline scenario of the Environmental Outlook of the Organization for Economic Cooperation and Development^54^, the Global Orchestration scenario of the MEA^28^ and the A1 scenario of IPCC-SRES^55^.

### DPPS model description

DPPS is a simple two-pool P-model^56^ including labile and stable soil pools and long-term P input and output data^15^. The DPPS model reproduces historical grass uptake as a function of P inputs (fertilizer and manure).

P inputs are allocated to two dynamic P pools, namely the stable (*P*_S_; 20%) and the labile P pools (*P*_L_; 80%). The model simulates the P transfers between the pools, the uptake of P by the grass and the size of both pools. To calculate the dynamics of P in these two pools, two differential equations are used:


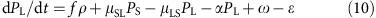



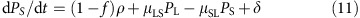


The rates of P transfer from *P*_L_ to *P*_S_ and vice versa are denoted by *μ*_LS_ and *μ*_SL_, respectively (per year). The coefficient *ρ* refers to the total P input (mineral fertilizer and manure). The coefficients *f* and 1−*f* refer to the fraction of *ρ* that transfers to *P*_L_ and *P*_S_, respectively. Coefficient *α* represents the grass P uptake fraction from *P*_L_. Parameters *ω* and *δ* are weathering and deposition inputs to *P*_L_ and *P*_S_, respectively. The parameter 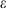 stands for soil P erosion-runoff outflow from *P*_L_. A large *μ*_LS_ makes *P*_L_ less available for plant uptake and a large *μ*_SL_ indicates that the stable pool acts as a buffer that replenishes the labile pool.

This model considers the essential P fluxes between grass and soil with a yearly time step. It can calculate P transfer between the labile and stable soil P pools, the P uptake by grass and the pool sizes. The model can also be formulated in a target-oriented approach^57^, in which the (future) P uptake is a model input and the required P application a result assuming no change in grassland area.

The DPPS model successfully simulated the historical patterns of crop P uptake as a response to the application rates in all continents and the entire globe as shown by Sattari *et al*.^15^ In the present paper, the model was used to calculate future P fertilizer and manure application rates in grasslands based on target grass productions in 2050 derived from the Rio+20 Trend (baseline) scenarios^21^. For more details of the model see Sattari *et al*.^15^ and Wolf *et al*.^56^

### Comparing the results with other studies

The estimates of global P budgets of grassland soils and P transfers between grasslands and croplands are compared in Supplementary Table 5, with those reported by other authors. Although it is not easy to compare all results obtained in this study with those in other studies, due to differences in approaches, system boundaries and the scope, a comparison of selected flows calculated here at country level with other studies is possible (Supplementary Table 5). Frequently, in literature larger values than the ones reported here are found, primarily due to inclusion of pigs and poultry, while in our study only ruminants are considered.

### Sensitivity analysis

The sensitivity of the results to 26-model parameters was investigated for 6 output variables for the soil P budget model, and the sensitivity of two output variables of the DPPS model to variation of 13 model parameters was investigated. These output variables represent global results for P uptake by grass (Supplementary Table 6). To limit computational load in the sensitivity analysis, the Latin Hypercube Sampling (LHS) technique^58^ was used. LHS offers a stratified sampling method for the separate input parameters, based on subdividing the range of each of the *k* parameters into disjunct equiprobable intervals based on a uniform distribution. The intervals were selected on the basis of earlier analysis of the livestock system. By sampling one value in each of the *N* intervals according to the associated distribution in this interval, we obtained *N* sampled values for each parameter. The number of runs *N* was 500 for the soil budget model and for the DPPS model.

The sampled values for the first model parameter are randomly paired to the samples of the second parameter, and these pairs are subsequently randomly combined with the samples of the third source and so on. This results in an LHS consisting of *N* combinations of *k* parameters. The parameter space is thus representatively sampled with a limited number of samples.

LHS can be used in combination with linear regression to quantify the uncertainty contributions of the input parameters to the model outputs^58^,^59^. The output *Y* considered (see columns in Supplementary Tables 7 and 8) is approximated by a linear function of the parameters *X*_*i*_ expressed by


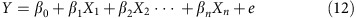


where *β*_*i*_ is the so-called ordinary regression coefficient and *e* is the error of the approximation. The quality of the regression model is expressed by the coefficient of determination (*R*^2^), representing the amount of variation *Y* explained by *Y*−*e*. Since *β*_*i*_ depends on the scale and dimension of *X*_*i*_, we used the standardized regression coefficient (SRC), which is a relative sensitivity measure obtained by rescaling the regression equation on the basis of the s.d.'s *σ*_*Y*_ and 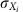:


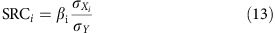


SRC_*i*_ can take values in the interval [−1, 1]. SRC is the relative change Δ*Y*/*σ*_*Y*_ of *Y* due to the relative change Δ*X*_*i*_/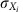 of the parameter *X*_*i*_ considered (both with respect to their s.d. *σ*). Hence, SRC_*i*_ is independent of the units, scale and size of the parameters. A positive SRC_*i*_ value indicates that increasing a parameter value will cause an increase in the calculated model output, while a negative value indicates a decrease in the output considered caused by a parameter increase.

The sum of squares of SRC_*i*_ values of all parameters equals the coefficient of determination (*R*^2^), which for a perfect fit equals 1. Hence, SRC_*i*_^2^/*R*^2^ yields the contribution of parameter *X*_*i*_ to *Y*. For example, a parameter *X*_*i*_ with SRC_*i*_=0.1 adds 0.01 or 1% to *Y* in case *R*^2^ equals 1.

Here we discuss the SRC for 26 parameters in the soil P budget model (Supplementary Table 7) and 13 parameters in the DPPS model (Supplementary Table 8). We focus on significant SRC values exceeding 0.2, which we consider to be an important influence on the model sensitivity or an important parameter (that is, a contribution of 0.2^2^=0.04 or about 4% to the variation of global results). Results for a similar analysis on the regional or smaller scale or different year would yield different results, depending on the P balances and local production system.

### Soil budget model

The fraction of the livestock production in mixed and industrial systems (FrProdint) is very important for both P uptake in 2005 and cumulative P uptake for 1970 and 2005 in mixed respectively. pastoral systems, but not in the aggregated livestock system (Supplementary Table 7). Excretion rates and the number of animals (LPN) are important for uptake in 2005 and cumulative uptake, in each system (mixed and pastoral) and in the aggregated livestock system.

The manure deposited in grasslands, the amount that is diverted outside agricultural systems and the manure used as organic fertilizer in grasslands (represented by ‘FrGrazing', ‘FrOthers' and ‘FrGrass', respectively) are not significant and not important for the sensitivity of uptake and cumulative uptake.

The kg meat or by-products per kg live weight (LWF), the DP—the ratio live weight to carcass—and the P contents of the various body parts (FPC) are all important and significant for uptake in 2005 and cumulative uptake in mixed systems and the aggregated livestock system, and for uptake in 2005 in pastoral systems. The parameter LWF is the fraction of each animal ‘product': meat; bones; blood; and so on. Therefore, when LWF increases, P in products and thus the extraction will be higher (SRC positive). Higher values of DP lead to a lower amount of P found in the (by) products and hence less uptake. Livestock production is not only used in equation (1) but also in the calculation of feed (equation (8)). The higher the production induces an increase of feed use, since it is linked to productivity. Therefore, the increase in feed can exceed the increase in the P in products leading to a decrease in uptake (since uptake=P in products+P in manure–P in feed).

### Dynamic Phosphorus Pool Simulator

Median simulated P uptake of global grasslands in 2005 is 15 Tg, with a variation of 11.0–17.6 Tg (2.5 and 97.5 percentile) as a result of variation of all the parameters in Supplementary Table 8. The cumulative P uptake (1970–2005) is 374–598 Tg around the median of 510 Tg. *U*_U0_ (initial P uptake from unfertilized soil) and *U*_F0_ (initial P uptake from fertilized soil) are the most important parameters for the sensitivity of all 13 parameters tested. *U*_U0_, *U*_F0_, *α* and *β* are all fitting parameters used to achieve the values for the size of LP and SP in year 0 of the simulation. *U*_U0_ (SRC=0.44; indicating sensitivity of 20%) and *U*_F0_ (SRC=0.64, indicating sensitivity of 41%) have a strong influence on the cumulative uptake of the total livestock system, and a smaller influence on the sensitivity of the uptake in 2005. The coefficient *C*_u_, the maximum fraction of crop P uptake from the labile pool is an important factor for the sensitivity of the cumulative uptake but has a smaller influence on the instantaneous uptake in 2005.

P input (*ρ*; manure plus mineral) has a stronger influence on the sensitivity of the instantaneous uptake in 2005 than on that of the cumulative uptake. This is due to depletion of the P in the two pools, which results in a strong and growing instantaneous effect of fertilizer, which is mostly flowing into the labile pool from which uptake occurs.

The time constant of P transfer from labile to stable pool, *μ*_LS_, is more important for the uptake in 2005 and cumulative uptake than *μ*_SL_, because the transfer from the labile to the stable pool is much faster, so variation of *μ*_LS_ has a much larger impact on uptake in 2005 and cumulative uptake than *μ*_SL_. Sensitivity to erosion (*ɛ*) is not significant. This is related to the initialization of the model when the initial uptake is determined by tuning the size of the slow and labile soil P pools to achieve an equilibrium and initial uptake, after this initialization erosion is no longer important because its value is small and it is almost constant. Sensitivity to the weathering rate is significant, but with a small influence.

## Additional information

**How to cite this article:** Sattari, S. Z. *et al*. Negative global phosphorus budgets challenge sustainable intensification of grasslands. *Nat. Commun.* 7:10696 doi: 10.1038/ncomms10696 (2016).

## Supplementary Material

Supplementary Information Supplementary Figures 1-4, Supplementary Tables 1-8 and Supplementary References

## Figures and Tables

**Table 1:** Grassland system P budget in 1970 and 2005.

**Region**	**Area (10**^**6**^** ha)**		**Grass P uptake (kg ha**^**−1**^**)**		**Internal manure (kg P per ha)**		**External manure (kg P per ha)**		**Fertilizer P (kg P per ha)**		Imported feed* **(kg P per ha)**		Exported manure to cropland* **(kg P per ha)**		**Livestock products (kg P per ha)**		**Other use of manure (kg P per ha)**		**Erosion (kg P per ha)**		**Grassland P budget**† **(kg P per ha)**	
	**1970**	**2005**	**1970**	**2005**	**1970**	**2005**	**1970**	**2005**	**1970**	**2005**	**1970**	**2005**	**1970**	**2005**	**1970**	**2005**	**1970**	**2005**	**1970**	**2005**	**1970**	**2005**
Africa	882	904	1.9	3.1	1.6	2.6	0.0	0.0	0.1	0.1	0	0.1	0.3	0.5	0.0	0.1	0.0	0.0	0.1	0.2	**−0.1**	**−0.4**
Asia	874	971	5.1	6.4	1.8	2.1	0.1	0.2	0.1	0.1	0.2	0.7	1.2	1.8	0.1	0.4	2.2	2.5	0.2	0.2	**−3.1**	**−3.7**
East Europe	107	115	10.2	4.0	8.0	3.4	0.7	0.6	0.4	0.1	2.6	2.2	3.1	1.4	1.2	0.8	0.3	0.1	0.6	0.6	**−1.3**	**0.2**
Latin America	487	546	4.0	6.0	3.6	4.9	0.0	0.0	0.1	0.1	0.2	0.2	0.5	1	0.2	0.4	0.0	0.0	0.3	0.5	**−0.5**	**−1.4**
North America	263	256	5.1	4.1	4.5	3.4	0.4	0.5	0	0	1.2	1.9	1.2	1.7	0.5	0.7	0.1	0.2	0.3	0.4	**−0.3**	**−0.4**
Oceania	462	407	1.3	1.4	1.2	1.2	0.0	0.0	0.1	0.1	0	0.1	0.1	0.1	0.1	0.1	0.0	0.0	0.1	0.1	**0.0**	**0.1**
West Europe	79	67	18	15.7	13.3	13.2	1.6	2.5	3.2	1.7	2.1	4	4.5	3.6	2.2	2.8	0.0	0.0	1.5	2.1	**−1.1**	**−0.1**
World	3,150	3,270	4.0	4.6	2.6	2.9	0.1	0.2	0.1	0.1	0.3	0.6	0.8	1.1	0.2	0.4	0.6	0.8	0.2	0.3	**−1.1**	**−1.5**
Intensive	560	537	13.2	16.1	7.6	8.8	0.6	1.1	0.6	0.4	1.9	3.5	4	5.8	1.0	1.6	2.4	3.4	0.7	1.0	**−4.8**	**−6.6**
Pastoral	2,590	2,730	2.0	2.4	1.5	1.7	0.1	0.2	0	0	0	0	0.1	0.2	0.1	1.1	0.2	0.3	0.1	0.2	**−0.2**	**−1.4**

Internal+external manure+fertilizer P are soil P inputs; grass P uptake is an output term in the soil P budget.

^*^Imported feed P represents the feed produced in croplands that is taken in by ruminants in the grassland system. Exported manure to cropland is the manure P produced by ruminants (intensive and pastoral) that is transferred to croplands.

^†^The grassland system P budget is computed as follows: external manure (applied manure from non-ruminants), fertilizer P, imported feed and atmospheric deposition (0.2 kg P per ha) are grassland P inputs; and export manure to cropland, livestock products' P, the other use of manure P and erosion are grassland P outputs. Weathering is an internal flow.

**Table 2:** Global manure P allocation in intensive and pastoral grassland systems in 1970 and 2005.

**Manure P allocation per source (Tg P per year)**	**World intensive**		**World pastoral**		**World total**		**Reference**
	**1970**	**2005**	**1970**	**2005**	**1970**	**2005**	
Total manure (grassland-based)	7.9	9.7	5.0	6.1	12.9	15.8	Equation (3)
Other use of manure outside the agricultural domain (manure use as fuel, building material and so on)	1.4 (17%)	1.8 (19%)	0.6 (13%)	0.7 (12%)	2.0 (16%)	2.6 (16%)	Equation (4)
Internal recycling of manure	**4.3 (54%)**	**4.7 (49%)**	**4.0 (80%)**	4.9 (80%)	**8.3 (64%)**	**9.6 (61%)**	Equations (5) and (6)
Export of manure (grassland-based manure application to croplands)	2.2 (29%)	3.1 (32%)	0.4 (7%)	0.5 (8%)	2.6 (20%)	3.5 (23%)	Equation (7)
Manure application from non-ruminants	**0.3**	**0.6**	**0.1**	**0.0**	**0.3**	**0.6**	External manure
Manure input to grassland soils (manure application and grazing in grasslands)	**4.6**	**5.3**	**4.1**	4.9	**8.6**	**10.2**	Sum of internal and external manure

**Table 3:** Abbreviations used in the soil P budget model, description and units.

**Symbol**	**Description**	**Units**
*y*	Year	Year
*c*	Country	—
*s*	Production systems	—
*a*	Animal category	—
*i*	Fraction of meat and by-products	—
*R*	Regional data	
PLMe	Phosphorus in meat and livestock by-products	kg P
LPD	Livestock production data	kg carcass
DP	Dressing percentage	kg carcass per kg live weight
LWF	Live weight partitioning fraction	kg fraction (muscle, adipose tissue and so on) per kg live weight
FPC	Phosphorus content	kg P per kg products or by-products
PLMi	Phosphorus in milk	kg P
MPD	Milk production data	kg milk
MPC	Milk phosphorus content	kg P per kg milk
Manure	Total P in manure excreted by livestock	kg P
LPN	Livestock population numbers	heads
Excretion rate	Annual P excretion for each animal category	kg N per head per year
Other uses	Amount of P in the manure allocated to other uses	kg P
FrOthers	Fraction of total manure allocated to other uses	—
Grazing	Amount of P in the manure allocated to grazing	kg P
FrGrazing	Fraction of total manure allocated to grazing	—
Grasslands	Amount of P in the manure that is applied in grasslands as fertilizer	kg P
FrGrass	Fraction of stored manure that is applied in grasslands as fertilizer	—
Croplands	Amount of P in the manure that is used in croplands as fertilizer	kg P
PFE	Total amount of phosphorus in livestock feed	kg P
LPR	Livestock productivity. It is calculated as the total amount of products associated with the animal category (carcass weight or milk) over the total number of animals	kg carcass per head (non-dairy cattle and sheep & goats) kg milk per head (dairy cattle)
LPN	Livestock population numbers	No. of heads
FEED	Total amount of feed item used in a certain region	kg feed item
PFI	Phosphorus content for a given feed item	kg P per kg feed item
PGU	Total grass phosphorus uptake	kg P

**Table 4:** Description of phosphorus flows, the animal categories involved and the data source.

**Flow**	**Description**	**Animal category**	Data source*
Products	Amount of P that leaves the grassland systems through animal products, for example, meat and milk, and by-products	Non-dairy cattle, dairy cattle and sheep and goats	FAOSTAT, literature
Livestock excretion	Total amount of P in grassland-based livestock excretion	Non-dairy cattle, dairy cattle, buffaloes, sheep and goats, horses, asses, mules and camels	IMAGE, FAOSTAT
Internal manure input	Amount of P in grassland-based livestock excretion that is returned to grassland soils	Non-dairy cattle, dairy cattle, buffaloes, sheep and goats, horses, asses, mules and camels	IMAGE, FAOSTAT
Spreading in cropland	Amount of P in grassland-based livestock excretion that is used as organic fertilizer in croplands	Non-dairy cattle, dairy cattle, buffaloes, sheep and goats, horses, asses, mules and camels	IMAGE
Other uses	Amount of P in grassland-based livestock excretion allocated to non-agricultural uses (fuel, building purposes and so on)	Non-dairy cattle, dairy cattle, buffaloes, sheep and goats, horses, asses, mules and camels	IPCC^60^, IMAGE
External manure input	Amount of P in non-grassland-based livestock excretion	Pigs and poultry	IMAGE, FAOSTAT
Livestock feed	Amount of P in crops used as feed for grassland-based livestock	Non-dairy cattle, dairy cattle, sheep and goats	FAOSTAT, literature, IMAGE
Grass uptake†	Amount of P that grass takes from grassland soils	Non-dairy cattle, dairy cattle, sheep and goats	Own calculations
Grass intake†	Amount of P in the grass used as feed for grassland-based livestock	Non-dairy cattle, dairy cattle, sheep and goats	Own calculations
Fertilization	Amount of P applied to grassland soils through mineral fertilization	NA	FAOSTAT, IFA, IMAGE
Erosion	Amount of P that is lost from grassland soils due to erosion and runoff	NA	Literature, IMAGE
Weathering, atmospheric deposition	Amount of P from weathering and atmospheric deposition	NA	Literature

NA, not applicable

^*^This table provides an indication where data have been obtained. For most agricultural data this is the FAO database FAOSTAT^22^, and in the IMAGE model these data have been stored as country-scale data and aggregated to the scale of world regions. Where IMAGE is the primary data source, it is actually obtained and or modified from Bouwman *et al*.^23^ It is impossible to provide the level of detail needed to recalculate individual numbers for individual countries, as this would require large matrices of data tables. Instead, the data tables are available electronically, together with the executables of the soil budget and DPPS model.

^†^Grass uptake and grass intake are assumed to be equal in the model, that is, losses of mown grass are neglected.
