# Supplementary material for: Negative global phosphorus budgets challenge sustainable intensification of grasslands
Source: Nat Commun. 2016 Feb 16;7:10696. doi: 10.1038/ncomms10696 (PMC4757762; doi:10.1038/ncomms10696)
Supplement: Supplementary Information — Supplementary Figures 1-4, Supplementary Tables 1-8 and Supplementary References [file ncomms10696-s1.pdf]

Negative global phosphorus budgets challenge sustainable intensification of grasslands

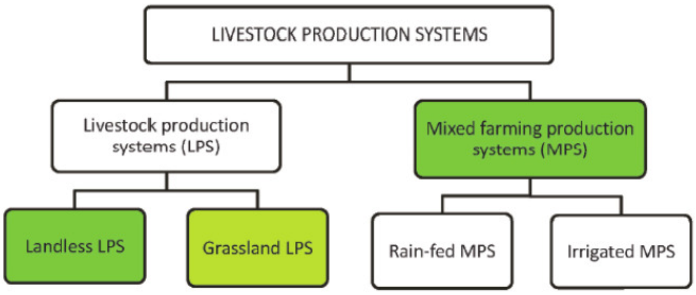


**a**

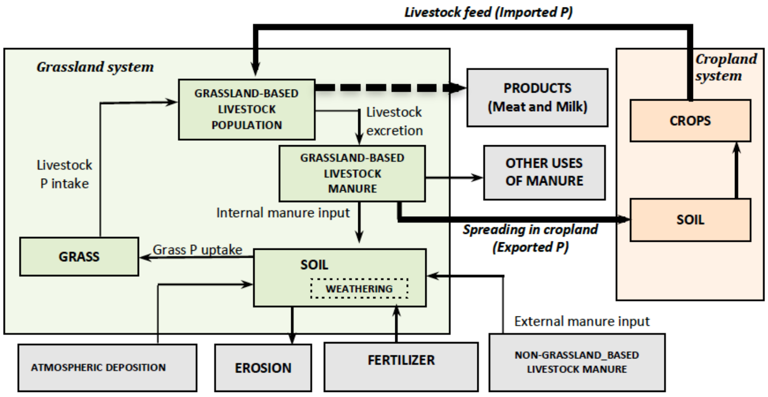


**b**

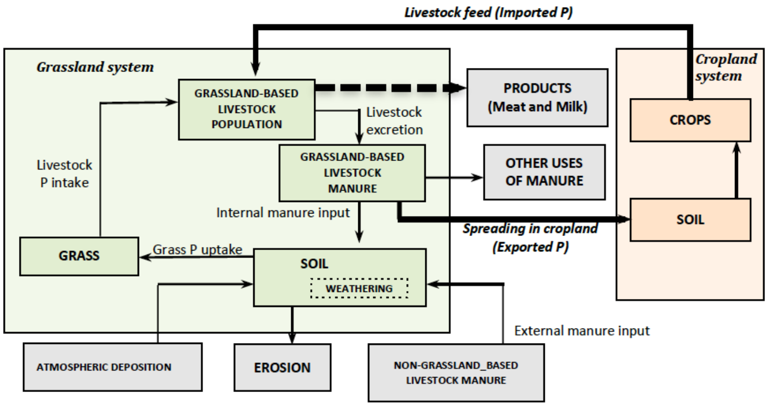


**c**

**Supplementary Figure 1| Livestock production systems and grassland systems classification.** The livestock production systems classification was adopted from Sere and Steinfeld [1] (**a**). Landless livestock and mixed farming production systems (dark green boxes in panel **a**) constitute the base of those intensive livestock systems characterized by important feed and manure exchange with croplands (**b**). Pastoral systems (**c**) are based on entirely grassland-based (GB) livestock production systems (light green in **a**) with negligible exchange of feed with cropland.

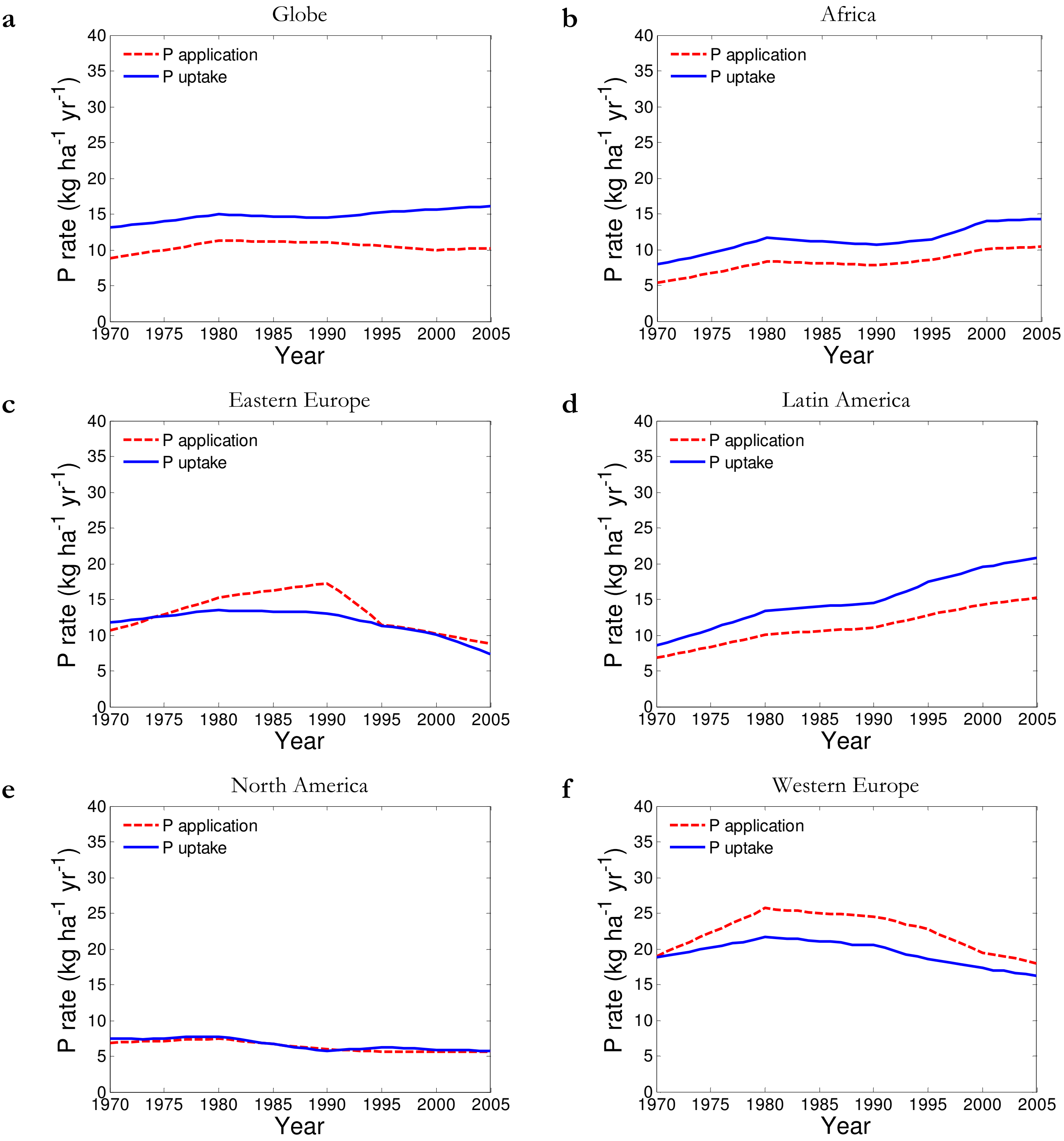


**Supplementary Figure 2| Intensive grassland system**. Historical trends of annual P application and P uptake in intensive grassland systems for the period 1970-2005 in (**a**) Globe, (**b**) Africa, (**c**) Eastern Europe, (**d**) Latin America, (**e**) North America, and (**f**) Western Europe. Dashed and solid lines represent P application and P uptake, respectively.

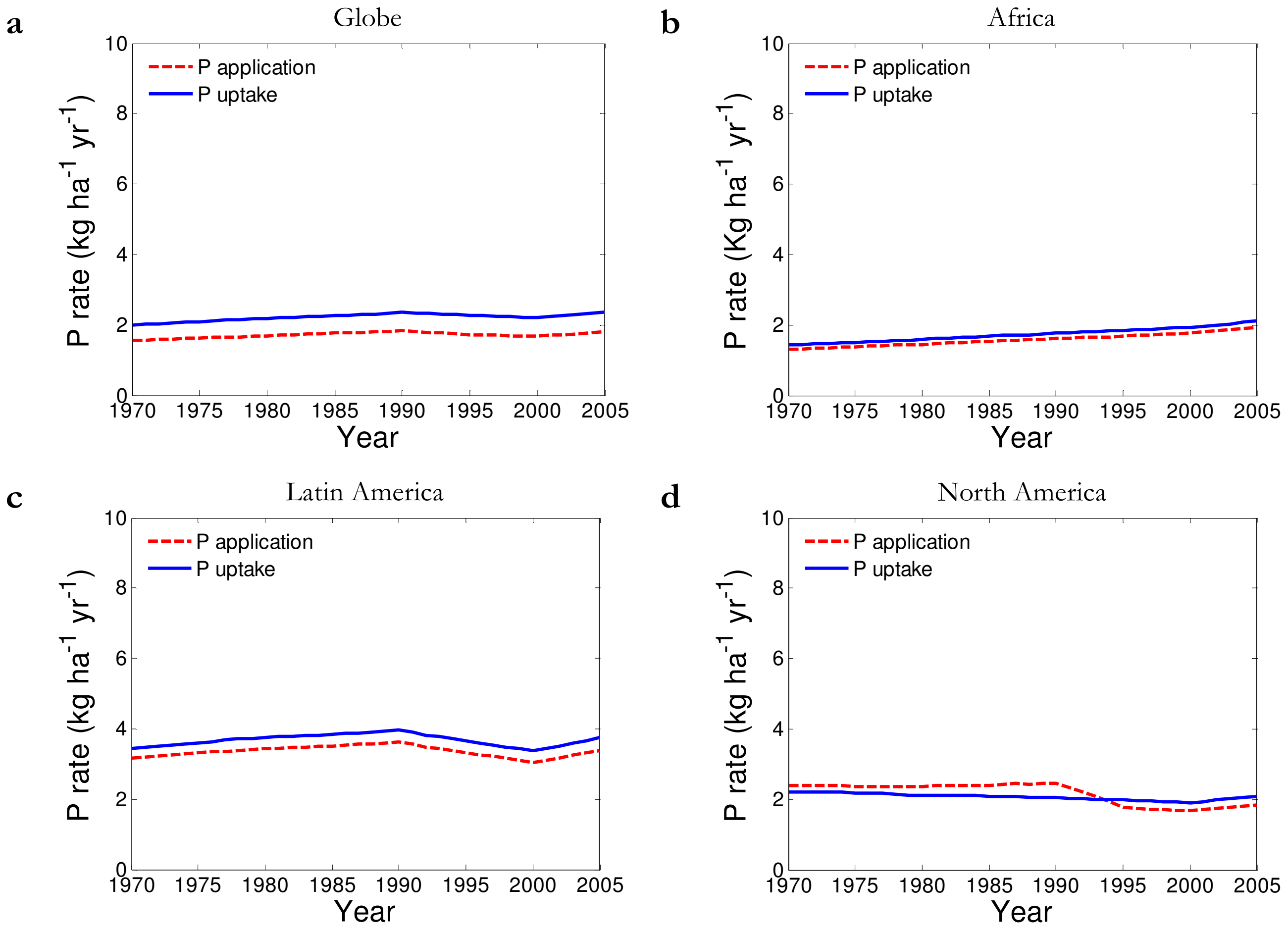


**Supplementary Figure 3| Pastoral grassland systems**. Historical trends of annual P application and P uptake in pastoral grassland systems for the period 1970-2005 in (**a**) Globe, (**b**) Africa, (**c**) Latin America, and (**d**) North America. Dashed and solid lines represent P application and P uptake, respectively. There are no pastoral systems in Europe.

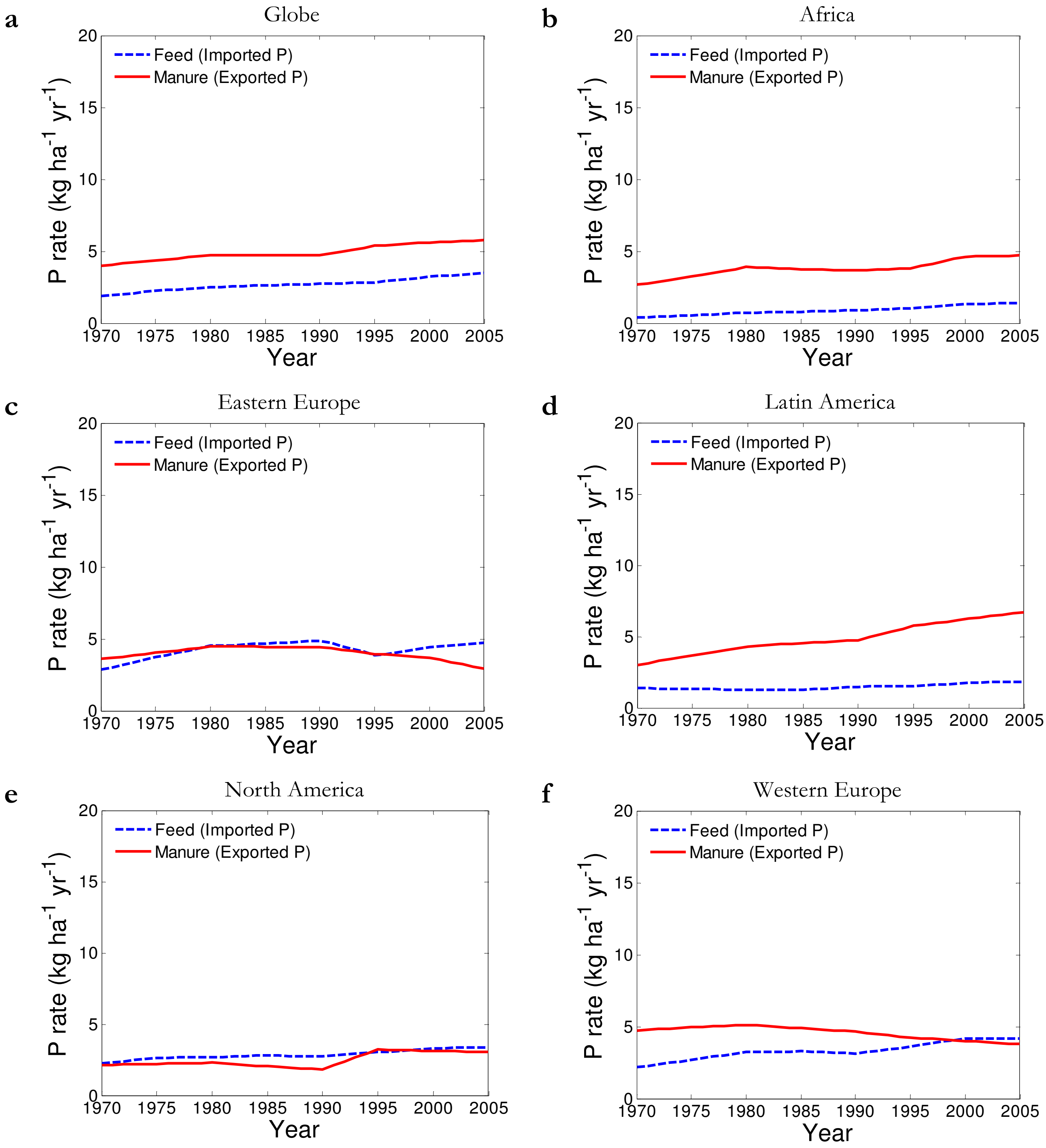


**Supplementary Figure 4| Intensive grassland system.** Historical trends of annual P import to and export from grasslands as livestock feed and manure applied to croplands, respectively, for the period 1970-2005 in (**a**) Globe (**b**) Africa, (**c**) Eastern Europe, (**d**) Latin America, (**e**) North America, and (**f**) Western Europe. Results for Asia and Oceania are not shown since the data for these two regions cannot be disaggregated.

**Supplementary Table 1|** List of countries and regions as used in the present study based on IMAGE [2,3]

| | |
|---|---|
| **AFRICA** | Algeria, Angola, Benin, Botswana, Burkina Faso, Burundi, Cameroon, Cape Verde, Central African Republic, Chad, Comoros, Congo, Côte d'Ivoire, Djibouti, Egypt, Equatorial Guinea, Eritrea, Ethiopia, Gabon, Gambia, Ghana, Guinea, Guinea Bissau, Kenya, Lesotho, Liberia, Libyan Arab Jamahiriya, Madagascar, Malawi, Mali, Mauritania, Mauritius, Morocco, Mozambique, Namibia, Niger, Nigeria, R,union, Rwanda, Sao Tome and Prince, Senegal, Seychelles, Sierra Leone, Somalia, South Africa, Sudan, Swaziland, Tanzania, The Democratic Republic of the Congo, Togo, Tunisia, Uganda, Western Sahara, Zambia, Zimbabwe. |
| **ASIA** | Afghanistan, Armenia, Azerbaijan, Bahrain, Bangladesh, Bhutan, Brunei Darussalam, Cambodia, China, Cyprus, Democratic People's Republic of Korea, Georgia, India, Indonesia, Iran, Iraq, Israel, Japan, Jordan, Kazakhstan, Kuwait, Kyrgyzstan, Lao People's Democratic Republic, Lebanon, Malaysia, Maldives, Mongolia, Myanmar, Nepal, Oman, Pakistan, Philippines, Qatar, Republic of Korea, Saudi Arabia, Singapore, Sri Lanka, Syrian Arab Republic, Taiwan, Tajikistan, Thailand, Timor-Leste, Turkey, Turkmenistan, United Arab Emirates, Uzbekistan, Vietnam, Yemen. |
| **LATIN AMERICA** | Antigua and Barbuda, Argentina, Bahamas, Barbados, Belize, Bermuda, Bolivia, Brazil, British Virgin Islands, Cayman Islands, Chile, Colombia, Costa Rica, Cuba, Dominica, Dominican Republic, Ecuador, El Salvador, Falklands Islands, French Guyana, Grenada, Guadeloupe, Guatemala, Guyana, Haiti, Honduras, Jamaica, Martinique, Mexico, Montserrat, Netherlands Antilles, Nicaragua, Panama, Paraguay, Peru, Puerto Rico, Saint Kitts and Nevis, Saint Lucia, Saint Vincent end the Grenadines, Suriname, Trinidad and Tobago, United States Virgin Islands, Uruguay, Venezuela. |
| **WESTERN EUROPE** | Albania, Austria, Belgium, Bosnia and Herzegovina, Croatia, Denmark, Estonia, Faroe Islands, Finland, France, Germany, Greece, Iceland, Ireland, Italy, Latvia, Lithuania, Luxemburg, Macedonia, Malta, Netherlands, Norway, Portugal, Slovenia, Spain, Sweden, Switzerland, United Kingdom. |
| **OCEANIA** | American Samoa, Australia, Cocos Islands, Cook Islands, Fiji, French Polynesia, Guam, Kiribati, Marshall Islands, Micronesia, Nauru, New Caledonia, New Zealand, Niue, Papua New Guinea, Samoa, Solomon Islands, Tokelau, Tonga, Tuvalu, Vanuatu, Wallis and Futuna |
| **EASTERN EUROPE** | Belarus, Bulgaria, Czech Republic, Hungary, Poland, Republic of Moldova, Romania, Russian Federation, Slovakia, Ukraine |
| **NORTH AMERICA** | Canada, Saint Pierre and Miquelon, United States of America |

**Supplementary Table 2|** Fractions and P content of live weight for meat and livestock by-products

| Animal category | Muscles | | Adipose tissue | | Skeleton | | Viscera | | Blood | | Skin & hair | | Gut content | |
|---|---|---|---|---|---|---|---|---|---|---|---|---|---|---|
| | % | P cont. | % | P cont. | % | P cont. | % | P cont. | % | P cont. | % | P cont. | % | P cont. |
| Non-dairy cattle | 31.3 | 212[a] | 19.8 | 730* | 13.5 | 950[b†] | 8.5 | 730[d] | 3.0 | 38[d] | 7.0 | 42[c] | 17.0 | 730* |
| Sheep & goats | 28.5 | 385[a] | 15.5 | 730* | 13.5 | 950[b†] | 11.0 | 730* | 4.0 | 38[d††] | 13.5 | 42[c‡] | 12.0 | 730* |

P content is expressed in mg P · $100g^{-1}$. All values of live weight fractions were obtained from [4]. Data on P content were obtained from (a) USDA [5], (b) Schalkwyk [6], (c) Silva ASd, et al [7] and (d) Williams et al. [8]. * Indicates that the numbers for adipose tissue, gut content and sheep & goats viscera were assumed to be equal to the P content in non-dairy cattle viscera. † Indicates that bone P content was assumed to be the same as the value for buffaloes found in Schalkwyk [6]. The blood content for sheep & goats was assumed (††) to be the same as the non-dairy cattle value. Similarly, P content for skin & hair in sheep & goats (‡) was taken from non-dairy cattle values.

**Supplementary Table 3|** Excretion rates for the different animal categories

| **Animal category** | **kg P head$^{-1}$ year$^{-1}$** |
|---|---|
| Beef cattle | 7.0 |
| Dairy cattle | 10.5 |
| Buffaloes | 7.0 |
| Pigs | 1.8 |
| Poultry | 0.1 |
| Sheep and goats | 1.4 |
| Horses | 6.5 |
| Asses | 4.3 |
| Mules | 4.3 |
| Camels | 7.9 |

**Supplementary Table 4** | Summary of manure allocation fraction values.

| Region | | Fraction other uses (***FrOther***) | | Fraction grazing (***FrGrazing***) | | Fraction application to grasslands (***FrGrass***) | |
|---|---|---|---|---|---|---|---|
| | | Intensive | Pastoral | Intensive | Pastoral | Intensive | Pastoral |
| **Africa** | Ave | 0.02 | 0.02 | 0.47 | 0.95 | 0.05 | 0.05 |
| **n* = 55** | Min | 0.00 | 0.00 | 0.15 | 0.93 | 0.01 | 0.01 |
| | Max | 0.18 | 0.18 | 0.65 | 0.95 | 0.05 | 0.05 |
| **Asia** | Ave | 0.36 | 0.35 | 0.27 | 0.80 | 0.05 | 0.05 |
| **n = 48** | Min | 0.00 | 0.00 | 0.10 | 0.50 | 0.01 | 0.01 |
| | Max | 0.70 | 0.70 | 0.50 | 1.00 | 0.25 | 0.25 |
| **Eastern Europe** | Ave | 0.00 | 0.00 | 0.44 | 1.00 | 0.40 | 0.40 |
| **n = 10** | Min | 0.00 | 0.00 | 0.40 | 1.00 | 0.25 | 0.25 |
| | Max | 0.00 | 0.00 | 0.50 | 1.00 | 0.50 | 0.50 |
| **Latin America** | Ave | 0.00 | 0.00 | 0.45 | 0.95 | 0.04 | 0.04 |
| **n = 44** | Min | 0.00 | 0.00 | 0.45 | 0.95 | 0.01 | 0.01 |
| | Max | 0.00 | 0.00 | 0.45 | 0.95 | 0.05 | 0.05 |
| **North America** | Ave | 0.13 | 0.07 | 0.33 | 1.00 | 0.50 | 0.50 |
| **n = 3** | Min | 0.10 | 0.00 | 0.19 | 1.00 | 0.50 | 0.05 |
| | Max | 0.18 | 0.10 | 0.40 | 1.00 | 0.50 | 0.25 |
| **Oceania** | Ave | 0.03 | 0.03 | 0.59 | 0.98 | 0.24 | 0.24 |
| **n = 22** | Min | 0.00 | 0.00 | 0.45 | 0.60 | 0.05 | 0.05 |
| | Max | 0.60 | 0.60 | 0.60 | 1.00 | 0.25 | 0.25 |
| **Western Europe** | Ave | 0.00 | 0.00 | 0.40 | 0.93 | 0.43 | 0.43 |
| **n = 29** | Min | 0.00 | 0.00 | 0.40 | 0.90 | 0.05 | 0.05 |
| | Max | 0.00 | 0.00 | 0.40 | 1.00 | 0.50 | 0.50 |

* n is the number of countries

**Supplementary Table 5 |** Phosphorus flows comparison with other studies

| Flow | Country | Year | Amount | This study (2005) |
|---|---|---|---|---|
| Products | France* [9] | 2002-2006 | 1.0 – 12.3 kg P $ha^{-1}$ | 2.5 kg P $ha^{-1}$ |
| | UK* [10] | 2009 | 32 Gg P $yr^{-1}$ | 21 Gg P $yr^{-1}$ |
| | Finland [11] | 1995-1999 | 4 Gg P $yr^{-1}$ | 3 Gg P $yr^{-1}$ |
| | US*[12] | 2007 | 390 Gg P $yr^{-1}$ | 157 Gg P $yr^{-1}$ |
| Animal excretion | France* [9] | 2002-2006 | 4.2 – 29.0 kg P $ha^{-1}$ | 17 kg P $ha^{-1}$ |
| | Finland [11] | 1995-1999 | 11 Gg P $yr^{-1}$ | 14 Gg P $yr^{-1}$ |
| Manure application to grassland | US* [12] | 2007 | 750 Gg P $yr^{-1}$ | 518 Gg P $yr^{-1}$ |
| Manure application to croplands | US* [12] | 2007 | 523 Gg P $yr^{-1}$ | 401 Gg P $yr^{-1}$ |
| Grass uptake/ intake | France* [9] | 2002-2006 | 2.8 – 11.3 kg P $ha^{-1}$ | 14.5 kg P $ha^{-1}$ |
| | UK* [10] | 2009 | 110 Gg P $yr^{-1}$ | 159 Gg P $yr^{-1}$ |
| Feed | France* [9] | 2002-2006 | 2.3 – 29 kg P $ha^{-1}$ | 3.2 kg P $ha^{-1}$ |
| | UK* [10] | 2009 | 95 Gg P $yr^{-1}$ | 26 Gg P $yr^{-1}$ |

*Ref. includes pigs and poultry

**Supplementary Table 6** | Model parameters included in the sensitivity analysis, the unit and the range for the sampling procedure.

| Parameter | Min. | Default | Max. | Type | Unit | Description |
|---|---|---|---|---|---|---|
| **Soil P budget model** | | | | | | |
| LPN | 0.9 | 1 | 1.1 | Multiplier | head | Number of animals by category |
| FrProdint | 0.75 | 1 | 1.25 | Multiplier | no dim | Fraction of the total production per category in mixed systems |
| Excretion rate | 0.75 | 1 | 1.25 | Multiplier | kg/animal/year | P excretion by animal category |
| Fertilizer | 0.9 | 1 | 1.1 | Multiplier | kg/year | P input from fertilizer + animal manure |
| FrGrazing | 0.75 | 1 | 1.25 | Multiplier | no dim | Fraction of the time spent by animals in animal houses |
| FrOthers | 0.75 | 1 | 1.25 | Multiplier | no dim | Fraction of the manure that ends outside the livestock system |
| FrGrass | 0.75 | 1 | 1.25 | Multiplier | no dim | Fraction of stored manure that is used outside the livestock system |
| LWF_ blood_cattle[a] | 0.75 | 1 | 1.25 | Multiplier | kg/kg | kg blood per kg live weight for cattle |
| LWF_bones_cattle[a] | 0.75 | 1 | 1.25 | Multiplier | kg/kg | kg bones per kg live weight for cattle |
| LWF_adipose_cattle[a] | 0.75 | 1 | 1.25 | Multiplier | kg/kg | kg adipose per kg live weight for cattle |
| LWF_gutcontent_cattle[a] | 0.75 | 1 | 1.25 | Multiplier | kg/kg | kg gut content per kg live weight for cattle |
| LWF_muscles_cattle[a] | 0.75 | 1 | 1.25 | Multiplier | kg/kg | kg muscles per kg live weight for cattle |
| LWF_skin_hair_cattle[a] | 0.75 | 1 | 1.25 | Multiplier | kg/kg | kg skin and hair per kg live weight for cattle |
| LWF_viscera_cattle[a] | 0.75 | 1 | 1.25 | Multiplier | kg/kg | kg viscera per kg live weight for cattle |
| LWF_ blood_sheepgoat[a] | 0.75 | 1 | 1.25 | Multiplier | kg/kg | kg blood per kg live weight for sheep and goats |
| LWF_bones_sheepgoat[a] | 0.75 | 1 | 1.25 | Multiplier | kg/kg | kg bones per kg live weight for sheep and goats |
| LWF_adipose_sheepgoat[a] | 0.75 | 1 | 1.25 | Multiplier | kg/kg | kg adipose per kg live weight for sheep and goats |
| LWF_gutcontent_sheepgoat[a] | 0.75 | 1 | 1.25 | Multiplier | kg/kg | kg gut content per kg live weight for sheep and goats |
| LWF_muscles_sheepgoat[a] | 0.75 | 1 | 1.25 | Multiplier | kg/kg | kg muscles per kg live weight for sheep and goats |
| LWF_skin_hair_sheepgoat[a] | 0.75 | 1 | 1.25 | Multiplier | kg/kg | kg skin and hair per kg live weight for sheep and goats |
| LWF_viscera_sheepgoat[a] | 0.75 | 1 | 1.25 | Multiplier | kg/kg | kg viscera per kg live weight for sheep and goats |
| DP | 0.75 | 1 | 1.25 | Multiplier | no dim | Dressing percentage (ratio carcass weight : live weight) |
| FPC[a] | 0.75 | 1 | 1.25 | Multiplier | kg/kg | P content in various body parts |
| Erosion | 0.75 | 1 | 1.25 | Multiplier | kg/year | Erosion |
| LPD | 0.75 | 1 | 1.25 | Multiplier | Gg | Production of milk and meat by animal category |
| FEED | 0.75 | 1 | 1.25 | Multiplier | Gg | Feed use by feed type by animal category |

| | | | | | | |
|---|---|---|---|---|---|---|
| **DPPS** | | | | | | |
| $C_u$ | 0.75 | 0.8 | 0.85 | Range | - | Coefficient to limit the uptake fraction from LP |
| μLS | 0.33 | 0.20 | 0.14 | Range | $year^{-1}$ | The rate of P transfer from labile to stable pool |
| μSL | 0.05 | 0.04 | 0.03 | Range | $year^{-1}$ | The rate of P transfer from stable to labile pool |
| $U_{U0}$ | 3.5 | 3.7 | 3.9 | Range | kg/ha/yr | Initial P uptake from unfertilized soil for initial pool size |
| $U_{F0}$ | 3.8 | 4 | 4.2 | Range | kg/ha/yr | Initial P uptake from fertilized soil for initial pool size |
| α | 1.1 | 1.2 | 1.3 | Range | no dim | Fitting parameter for initial pool size |
| β | 1.0 | 1.1 | 1.2 | Range | no dim | Fitting parameter for initial pool size |
| ω | 0.8 | 1 | 1.2 | Range | kg/ha/yr | Weathering rate |
| δ | 0.15 | 0.25 | 0.35 | Range | kg/ha/yr | Atmospheric deposition input to stabile pool |
| *f* | 0.7 | 0.8 | 0.9 | Range | kg | The coefficients f refers to the fraction of total P input (mineral fertilizer and manure) that transfers to labile pool |
| ε | 0,75 | 1 | 1.25 | Multiplier | kg/ha/yr | soil P erosion-runoff outflow from labile pool |
| ρ | 0,75 | 1 | 1.25 | Multiplier | kg/ha/yr | P input from fertilizer + animal manure |
| $U_i$ | 0,75 | 1 | 1.25 | Multiplier | kg/ha/yr | Grass uptake initial year |

[a] The fraction of the various components LWF had to be taken separately because the sum of the fraction has to be 1.0; The P contents can be varied for all body parts at the same time.

**Supplementary Table 7 |** Standardized regression coefficient (SRC)a values representing the relative sensitivity of 6 model output variables representing global model results for the grass P uptake in intensive and pastoral systems and the aggregated total for the year 2005 and for the cumulative total for 1970-2005 for the global soil budget model (columns) to variation in 26 parameters (rows, see Supplementary Table 6).[a]

| | **Intensive systems** | | **Pastoral systems** | | **Aggregated livestock system** | |
|---|---|---|---|---|---|---|
| **Parameter** | **upt_2005_int** | **upt_cum_int** | **upt_2005_ext** | **upt_cum_ext** | **upt_2005_tot** | **upt_cum_tot** |
| FrProdint | 0.63 | 0.58 | -0.76 | -0.72 | | 0,05 |
| Excretion rate | 0.66 | 0.69 | 0.62 | 0.66 | 0.91 | 0.91 |
| FrGrazing | | | | | | |
| FrOthers | | | | | | |
| FrGrass | | | | | | |
| Erosion | -0.01 | -0.01 | | | | 0.00 |
| LPN | 0.26 | 0.28 | 0.24 | 0.26 | 0.36 | 0.36 |
| Fertilizer | | | | | | |
| LWF_ blood_cattle | | | | | | |
| LWF_bones_cattle | | | | | | |
| LWF_adipose_cattle | | | | | | |
| LWF_gutcontent_cattle | | | | | | |
| LWF_muscles_cattle | | | | | | |
| LWF_skin_hair_cattle | | | | | | |
| LWF_viscera_cattle | | | | | | |
| LWF_ blood_sheepgoat | | | | | 0.00 | |
| LWF_bones_sheepgoat | | | -0.01 | -0.02 | 0.00 | |
| LWF_adipose_sheepgoat | | | | | | |
| LWF_gutcontent_sheepgoat | | | | | | |
| LWF_muscles_sheepgoat | | | | | | |
| LWF_skin_hair_sheepgoat | | | | | | |
| LWF_viscera_sheepgoat | | | | | | |
| FPC | 0.03 | 0.01 | 0.04 | 0.04 | 0.04 | 0.03 |
| DP | -0.03 | -0.02 | -0.03 | -0.03 | -0.04 | -0.03 |

| | | | | | | | |
|---|---|---|---|---|---|---|---|
| LPD | -0.11 | -0.10 | | | -0.09 | | -0.08 |
| FEED | 0.01 | 0.07 | | 0.02 | | | 0.06 |

[a] Cells in with no values represent insignificant SRC values; cells with values show significant SRC, grey colours indicate values -0.2<SRC < 0.2; green and salmon colours indicate values exceeding -0.2 and +0.2, respectively. An SRC value of 0.2 indicates that the parameter concerned has an influence of $0.2^2 = 0.04$ (4%) on the model variable considered.

**Supplementary Table 8 |** Standardized regression coefficient (SRC) values representing the relative sensitivity of 2 model output variables representing global model results for the DPPS model (columns) to variation in 13 parameters (rows, see Table Supplementary 6).[a]

| Parameter | uptake_2005 | uptake_cum |
|---|---|---|
| $C_u$ | 0.17 | **0.21** |
| $\mu LS$ | **0.22** | **0.27** |
| $\mu SL$ | 0.06 | 0.04 |
| $U_{U0}$ | **-0.40** | **-0.44** |
| $U_{F0}$ | **0.55** | **0.64** |
| $\alpha$ | | 0.06 |
| $\beta$ | **0.28** | **0.30** |
| $\omega$ | 0.15 | 0.11 |
| $\delta$ | | |
| $f$ | | |
| $\varepsilon$ | | |
| $\rho$ | **0.35** | 0.15 |
| $U_i$ | | |

[a] Cells in with no values represent insignificant SRC values; cells with values show significant SRC, grey colours indicate values -0.2<SRC < 0.2; green and salmon colours indicate values exceeding -0.2 and +0.2, respectively. An SRC value of 0.2 indicates that the parameter concerned has an influence of $0.2^2 = 0.04$ (4%) on the model variable considered.

## Supplementary References


1. Seré, C. & Steinfeld, H. *World livestock production systems. Current status, issues and trends*. (Food and Agriculture Organization of the United Nations, 1996).
2. Stehfest, E., Van Vuuren, D. P., Kram, T. & Bouwman, A. F. (PBL Netherlands Environmental Assessment Agency (http://themasites.pbl.nl/models/image/index.php/Main_Page), The Hague, . (2014).
3. Bouwman, A., Kram, T. & Klein Goldewijk, K. *Integrated modelling of global environmental change. An overview of IMAGE 2.4*. (Netherlands Environmental Agency (MNP), 2006).
4. Kempster, T., Cuthbertson, A. & Harrington, G. *Carcase evaluation in livestock breeding, production and marketing*. (Granada, 1982).
5. USDA. National_Agricultural_Statistics_Service. Quick Stats (ftp://ftp.nass.usda.gov/quickstats/). retrieved 21 March. (2015).
6. Schalkwyk, O. L. v. Bone density and calcium phosphorus content of the giraffe and african buffalo skeletons. (University of Pretoria, 2004).
7. Silva, A. S. d. *et al.* Characteristics of carcass and non-carcass components in feedlot native goas in the Brazilian semiarid region. *Revista Brasileria de Zootecnia* **40**, 1815:1821 (2011).
8. Williams, S. N., McDowell, L. R., Warnick, A. C., Wilkinson, N. S. & Lawrence, L. A. Phosphorus concentrations in blood, milk, feces, bone and selected fluids and tissues fo growing heifers as affected by dietary phosphorus. *Livestock Research for Rural Development* **3** (1991).
9. Senthilkumar, K., Nesme, T., Mollier, A. & Pellerin, S. Regional-scale phosphorus flows and budgets within France: The importance of agricultural production systems. *Nutrient Cycling in Agroecosystems* **92**, 145-159 (2012).
10. Cooper, J. & Carliell-Marquet, C. A substance flow analysis of phosphorus in the UK food production and consumption system. *Resources, Conservation and Recycling* **74**, 82-100 (2013).
11. Antikainen, R. *et al.* Stocks and flows of nitrogen and phosphorus in the Finnish food production and consumption system. *Agriculture, Ecosystems and Environment* **107**, 287-305 (2005).
12. MacDonald, G. K., Bennett, E. M. & Carpenter, S. R. Embodied phosphorus and the global connections of United States agriculture. *Environmental Research Letters* **7** (2012).
